# Astrocyte Kir4.1 expression level territorially controls excitatory transmission in the brain

**DOI:** 10.1016/j.celrep.2025.115299

**Published:** 2025-02-12

**Authors:** Olga Tyurikova, Olga Kopach, Kaiyu Zheng, Daman Rathore, Neela Codadu, Sheng-Yi Wu, Yi Shen, Robert E. Campbell, Rob C. Wykes, Kirill Volynski, Leonid P. Savtchenko, Dmitri A. Rusakov

**Affiliations:** 1 Department of Clinical & Experimental Epilepsy, UCL Queen Square Institute of Neurology, University College London, London WC1N 3BG, UK; 2 Department of Chemistry, University of Alberta, Edmonton, Alberta T6G 2G2, Canada; 3 Department of Chemistry, Graduate School of Science, The University of Tokyo, Tokyo 113-0033, Japan; 4 Division of Neuroscience & Centre for Nanotechnology in Medicine, The University of Manchester, Manchester M13 9PL, UK; 5 Neuroscience and Cell Biology Research Institute, City St George’s, University of London, Cranmer Terrace, London SW17 0RE, UK

## Abstract

Intense brain activity elevates extracellular potassium, potentially leading to overexcitation and seizures. Astrocytes are crucial for restoring healthy potassium levels, and an emerging focus on their Kir4.1 channels has reopened the quest into the underlying mechanisms. We find that the Kir4.1 level in individual astrocytes sets the kinetics of their potassium and glutamate uptake current. Combining electrophysiology with multiplexed optical sensor imaging and FLIM reveals that rises in extracellular potassium would normally boost presynaptic Ca^2+^ entry and release probability at excitatory synapses unless such synapses are surrounded by the Kir4.1-overexpressing astrocytes. Inside the territories of Kir4.1-overexpressing astrocytes, high-frequency afferent stimulation fails to induce long-term synaptic potentiation, and the high-potassium waves of cortical spreading depolarization are markedly attenuated. Biophysical exploration explains how astrocytes can regulate local potassium homeostasis by engaging Kir4.1 channels. Our findings thus point to a fundamental astrocytic mechanism that can restrain the activity-driven rise of excitability in brain circuits.

## Graphical abstract

**Graphical abstract:**
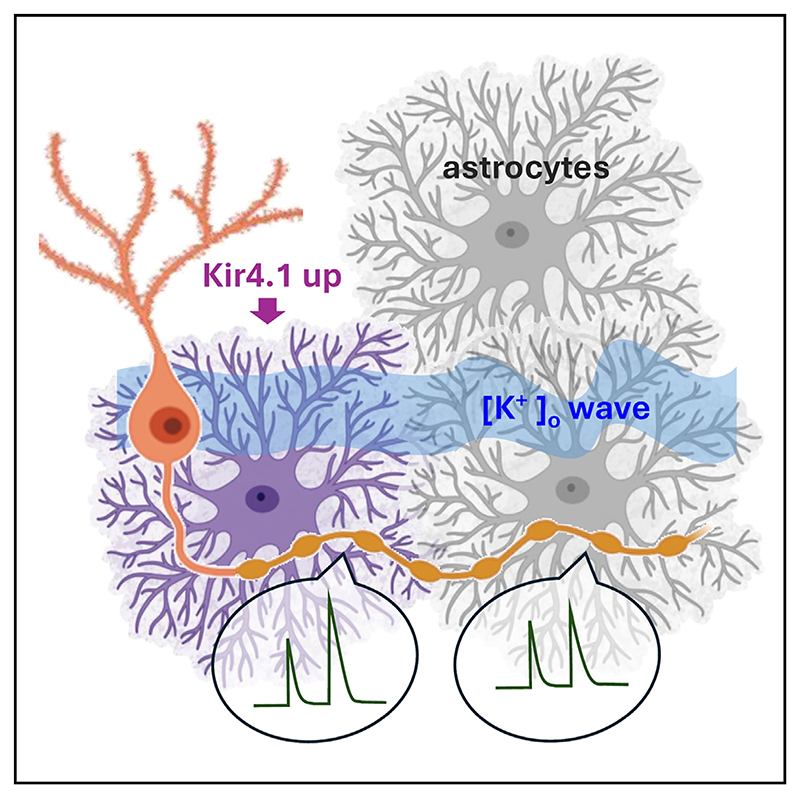

## Introduction

Intense neuronal activity can elevate the concentration of brain extracellular potassium ([K^+^]_out_) from its resting level of 2.5–3 mM to up to 10 mM,^1^ potentially upsetting the balance of neuronal and network excitability.^2^ Even greater [K^+^]_out_ elevations could occur in pathological conditions such as during cortical spreading depolarization^3,4^ or, at least in theory, in microscopic perisynaptic hotspots of K^+^ efflux.^5,6^ The critical role of maintaining healthy [K^+^]_out_ has long been associated with astrocytes.^7,8^ While these cells express multiple membrane mechanisms of K^+^ regulation,^9^ the focus has been on the Na,K-ATPase (NKA) pumps, the Na^+^/K^+^/Cl^−^ exchanger (NKCC1), and the inwardly rectifying K^+^ channel Kir4.1.^10–12^ When [K^+^]_out_ elevations were induced by sustained electrical stimulation (200–500 stimuli applied over 5–300 s), the key routes of K^+^ uptake pointed to the NKA^13^ and NKCC1.^14^ Further tests employing intense stimuli or direct high-[K^+^]_out_ application suggested a primary role for the NKA,^15^ although at the concentrations applied the non-specific NKA blocker ouabain could show off-target actions.^16^ However, the intense electrical stimulation of multiple afferents, which triggers severalfold increases in tissue-average [K^+^]_out_, is unlikely to represent a common physiological scenario. The high sensitivity of Kir4.1 current to [K^+^]_out_ near its resting level^17,18^ and the sharp enhancement of NKA function upon membrane depolarization^19,20^ are likely to change dramatically their respective contributions to K^+^ uptake during extreme [K^+^]_out_ elevations.^21^

In fact, in response to a single afferent stimulus, hippocampal astrocytes generate an inward (mainly K^+^) current peaking at 20–50 pA and lasting 5–10 s,^6,22,23^ thus carrying a charge in the region of ~2 × 10^−1^ coulomb, or approximately 10^9^ K^+^ ions. The cell volume of a typical hippocampal astrocyte is ~5 × 10^3^ μm^3^ (Bushong et al.^24^), which represents ~10% tissue volume fraction in the hippocampal neuropil.^25,26^ Because the extracellular space occupies approximately a twice greater (~20%) tissue volume fraction,^27–29^ an individual astrocyte territory corresponds to ~10^4^ μm^3^ of the extracellular lumen. An amount of 10^9^ K^+^ ions in 10^4^ μm^3^ volume represents a concentration of ~0.1 mM, which thus estimates the volume-average [K^+^]_out_ elevation range in response to one synchronous discharge of multiple afferents. This value appears in excellent correspondence with the [K^+^]_out_ increases evoked by a pair of afferent stimuli in the hippocampal area CA1, as measured with a K^+^-sensitive electrode.^30^

The conditional knockout of astrocytic Kir4.1^10–12^ inhibited key astrocyte functions including extracellular K^+^ buffering and glutamate uptake, leading to increased cell excitability and enhanced synaptic potentiation.^31–33^ Equipped with this genetic tool, an elegant electrophysiological study found that ~80% of astrocyte K^+^ current during individual afferent discharges was through Kir4.1 channels^23^: this observation helped to establish the Kir4.1 kinetics based on experimental recordings.^34^ It was also reported that blocking Kir-type channels slows down glutamate clearance by the astrocyte during repetitive afferent discharges in the frontal cortex.^35^ While these findings highlight deficiencies of neuronal communication in the absence of astrocyte Kir4.1, the question arises whether and how the variations in Kir4.1 expression among individual astrocytes could affect local synaptic circuitry. Indeed, a loss of function or reduced expression of Kir4.1 has been associated with neurode-generative disorders,^36^ such as hippocampal sclerosis,^37^ animal models of Huntington’s disease,^38,39^ Rett syndrome,^40^ fragile X syndrome,^41^ or epilepsy,^42,43^ whereas Kir4.1 overexpression has been related to depression behavior^44^ and appears as a compensatory mechanism under pilocarpine-induced seizures.^45^

In view of these important regulatory phenomena, the virus-driven overexpression of Kir4.1 in individual astrocytes^38,44,46^ has provided a principal tool for direct experimental comparison *in situ* between astrocytic environments with varied expression of Kir4.1. A recent study has found that overexpression of Kir4.1 reduced activity-dependent progression in the depolarization of perisynaptic astrocyte processes, thus also impacting local glutamate transport.^47^ Building on these discoveries, our aim was to understand whether, how, and under what conditions the expression level of Kir4.1 in individual astrocytes could regulate local synaptic signal transfer and its use-dependent plasticity. The results we have obtained provide a neurophysiological basis for considering astrocytic Kir4.1 expression as a potential target for therapeutic intervention.

## Results

### Kir4.1 overexpression hyperpolarizes astrocytes increasing their K^+^ conductance

**Figure 1:**
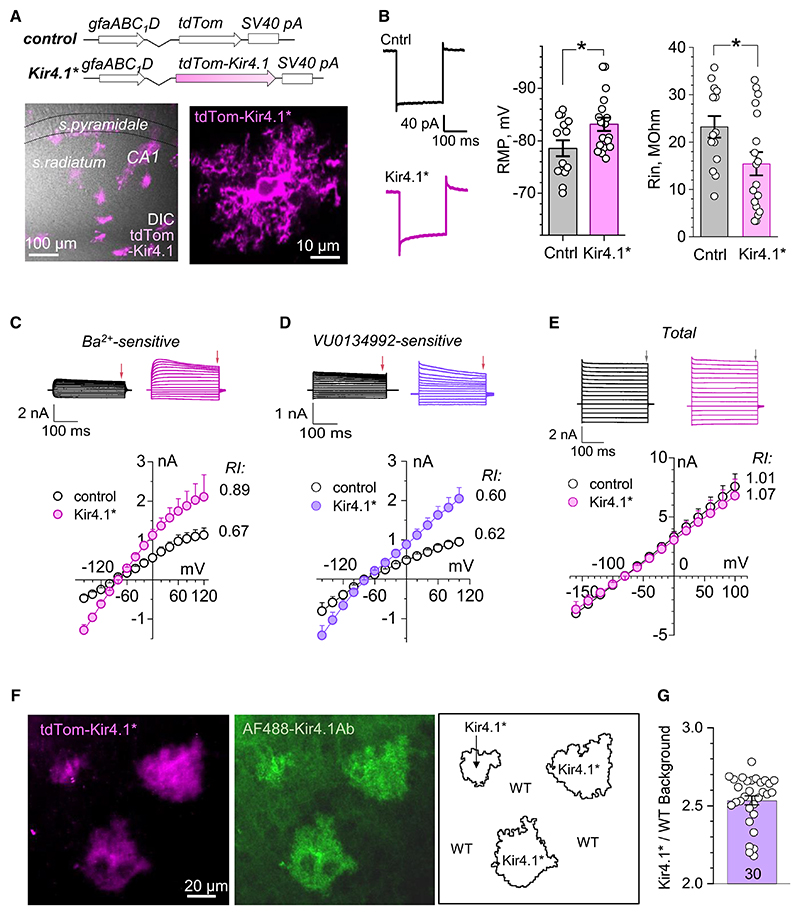
Kir4.1-overexpressing astrocytes display reduced input resistance and increased K^+^ currents (A) Schematics: AAV vectors have been designed to express, under the GFAP promoter, astrocyte-specific TdTomato (control) or overexpress TdTomato-labeled Kir4.1 channels (“Kir4.1* astrocytes”). Images: examples of Kir4.1 astrocytes in a hippocampal slice (left, general view; right, an individual cell, DIC, and TdTomato channels combined); λ_x_^2P^ = 940 nm. Scale bars, 100 μm (left) and 10 μm (right). (B) Traces: examples of whole-cell voltage-clamp recordings from control and Kir4.1* astrocytes, as indicated. Graphs: statistical summary (bars, mean ± SEM) for resting membrane potential (RMP, left) and input resistance (R_in_, right) in control (*n =* 14) and Kir4.1* astrocytes (*n =* 18), as indicated; dots, readouts from individual cells; *p < 0.05 (t test). (C) Current traces: representative examples of a voltage-step protocol for the current component sensitive to BaCl_2_ (200 μM). Graph: *I-V* curves (mean ± SEM) for control and Kir4.1* astrocytes, as indicated (*n =* 9 and 8, respectively); small arrow indicates voltage readout area; RI, rectification index. (D) Experiment as in (C), but for the current component sensitive to VU0134992 (40 μM; *n* = 8 and 8 for control and Kir4.1*, respectively); notations as in (C). (E) Experiment as in (C), but for the total astrocyte current (*n = 9* and 8 for control and Kir4.1*, respectively); notations as in (C). (F) Visualizing Kir4.1 expression in control (WT) and Kir4.1* astrocytes, one region of interest (ROI) example: left, red (shown as magenta) channel displaying Kir4.1* astrocytes; center, green channel displaying antibody-labeled Kir4.1; right, territories of Kir4.1* and WT astrocytes, as illustrated. Scale bar, 10 μm (applied throughout). (G) The Kir4.1*/WT fluorescence intensity ratios in the AF488-Kir4.1Ab channel for the territories around individual Kir4.1* astrocytes (bars, mean ± SEM) as illustrated in (F, right); circles, data for individual neighborhoods of Kir4.1* astrocytes; collected from three slices.

To express additional Kir4.1 channels in native astrocytes,^46^ we employed adeno-associated viruses of the 9 serotype under an astrocyte-specific promoter (gfaABC1D), delivering either TdTomato-tagged Kir4.1 channels (AAV-GFAP::TdTom-Kir4.1) or TdTomato alone (AAV-GFAP::TdTom; control) to individual astrocytes (Figure 1A, diagrams). Following unilateral intracere-broventricular injection in neonatal pups, at least 21 days elapsed, as described previously,^6,48^ to achieve moderately sparse expression at individual astrocytes (Figure 1A, image panels). Holding identified astrocytes whole cell, we found that overexpressing Kir4.1 shifted their resting membrane potential from −78.6 ± 1.5 mV (*n =* 14) to −83.2 ± 1.5 mV (*n =* 18; Figure 1B). This hyperpolarization is likely to occur because the increased Kir4.1 channel conductance would make the membrane potential closer to the (more negative) K^+^ equilibrium potential. The Kir4.1 overexpression also lowered cell-membrane input resistance, from 22.0 ± 2.5 MΩ (*n* = 14) to 15.4 ± 2.5 MΩ (*n* = 18; *p* < 0.05, *t* test; Figure 1B).

To test whether these changes could indeed be explained by the increased K^+^ conductance, we documented the I-V relationships for the Ba^2+^-sensitive current component commonly associated with K^+^ channel conductance. The Kir4.1-overexpressing astrocytes (termed “Kir4.1* astrocytes” thereafter) displayed an approximately 2-fold K^+^ current increase (Figure 1C). Since (passive) K^+^ conductance in hippocampal CA1 astrocytes is provided mainly by homomeric Kir4.1 but also heteromeric Kir4.1/5.1 channels^49^ we used the selective Kir4.1 antagonist VU0134992^50^ to dissect the contribution of Kir4.1. The application of VU0134992 (40 μM) confirmed a 2-fold increase in isolated K^+^ currents in Kir4.1* compared with control astrocytes (Figure 1D). Our recordings have also confirmed that Kir4.1 currents exhibit relatively weak rectification in CA1 hippocampal astrocytes in baseline conditions and are several times smaller than the total astrocyte currents, which displayed no detectable rectification^51^ (Figure 1E).

To determine to what degree the electrophysiological data represent overexpression levels of Kir4.1, we carried out immunostaining of Kir4.1 in the slices with a sparse presence of Kir4.1* astrocytes (Figure 1F; methods). The average fluorescence intensity ratio between inside and outside the territories of Kir4.1* astrocytes (Figure 1F, right panel) was 2.53 ± 0.03 (mean ± SEM, *n =* 30 cell neighborhoods; Figure 1G), thus providing a direct estimate of the Kir4.1 overexpression level in Kir4.1* astrocytes. This value appeared in good correspondence with the detected 2-fold increases in K^+^ currents in Kir4.1* cells compared with control.

### Increased Kir4.1 expression has no effect on astrocyte Ca^2+^ homeostasis but accelerates K^+^ and glutamate uptake currents

**Figure 2:**
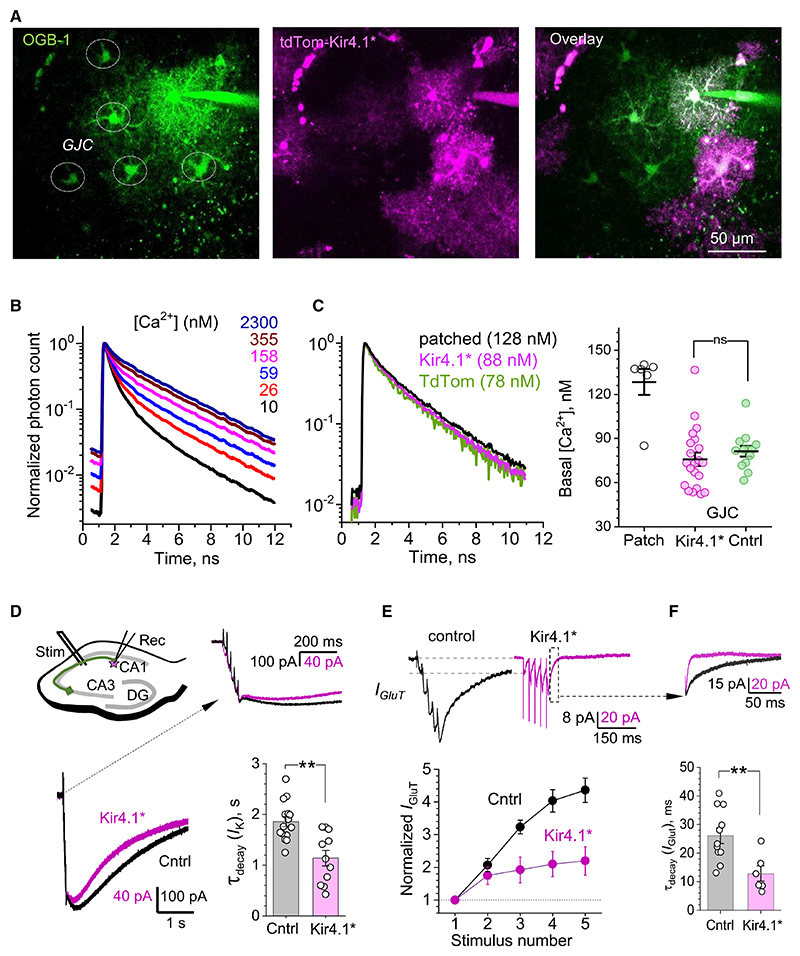
Kir4.1 overexpression accelerates astrocytic K^+^ and glutamate uptake currents but has no effect on [Ca^2+^] (A) Multiplexed imaging of astrocytes held whole cell and dialyzed with OGB-1, their gap-junction-connected (GJC) neighbors (left), TdTom-labeled Kir4.1* astrocytes (middle), and astrocytes under combined conditions (right); λ_x_^2P^ = 940 for TdTomato and λ_x_^2P^ = 800 nm for OGB-1. Scale bar, 50 mm (applied throughout). (B) FLIM calibration of OGB-1 sensitivity for [Ca^2+^]: fluorescence lifetime kinetics of OGB-1 in selected solutions of clamped [Ca^2+^], as indicated; see Zheng and co-workers^55,56^ for protocol detail and Figures S1A and S1B for full calibration data. (C) Examples of the OGB-1 fluorescence decay kinetics (FLIM) inside the patched, GJC Kir4.1*, and GJC TdTom astrocytes (control), as indicated. Graph: summary (bars, mean ± SEM) of the FLIM-based measurements of basal [Ca^2+^] in patched (*n* = 6), GJC Kir4.1* (*n* = 29), and GJC control (*n* = 13) astrocytes, as indicated; dots, individual cell readouts; see Figures S1C and S1D for further detail. (D) Schematic, experiment diagram. Traces: whole-cell astrocyte current evoked by 5 × 50 Hz stimuli applied to Schaffer collaterals in control (black) and Kir4.1* (pink) astrocytes, superimposed and normalized to the fifth stimulus peak, at two timescales as shown (arrow). Graph: summary of the decay time (mean ± SEM) for control and Kir4.1* astrocytes (*n* = 15 and 11, respectively), as indicated; dots, individual cell readouts; **p < 0.01 (*t* test). (E) Traces: examples of the glutamate transporter current (TBOA-sensitive, *I*_*GluT*_) in control (black) and Kir4.1* astrocytes (pink) current evoked by 5 × 50 Hz stimuli applied to Schaffer collaterals and rescaled to the first peak (dotted lines). Graph: summary (mean ± SEM) of the current amplitude progression in control and Kir4.1* astrocytes (*n* = 11 and 6, respectively) normalized to the first amplitude; **p < 0.01 (*t* test). (F) Traces: enlarged glutamate transporter current shown in (E), superimposed, and rescaled to the fifth stimulus peak. Graph: summary (bars, mean ± SEM) of the transporter current decay for control and Kir4.1* astrocytes (*n* = 11 and 6, respectively); dots, individual cell readouts; **p < 0.01 (two-way RM ANOVA).

Astrocytes communicate their intra- and extracellular physiological signals by engaging multimodal Ca^2+^ elevations,^52,53^ which are in turn regulated by their local resting Ca^2+^ concentration ([Ca^2+^]).^54^ To understand whether Kir4.1 overexpression alters astrocytic Ca^2+^ homeostasis, we patch-loaded astrocytes with the fluorescent Ca^2+^ indicator OGB-1: its fluorescence lifetime sensitivity to [Ca^2+^] enables scattering-independent FLIM monitoring of nanomolar range intracellular [Ca^2+^].^55^ Because inter-astrocyte gap junctions are permeable to OGB-1, the FLIM analysis of [Ca^2+^] can be carried out in gap-junction-connected astrocytes that are unperturbed by the pipette dialysis^56^ (Figure 2A). Once the [Ca^2+^] FLIM readout has been calibrated (Figures 2B, S1A, and S1B), we found no difference in the baseline [Ca^2+^] between Kir4.1* (*n =* 29) and control astrocytes (*n* = 13; Figures 2C, 2D, S1C, and S1D), or in the rates or magnitudes of spontaneous Ca^2+^ elevations (Figure S2), thus suggesting no major effects on Ca^2+^ homeostasis. As for the afferent stimulus-evoked astrocyte Ca^2+^ responses, their remarkably strong variability reported in numerous studies involves multiple (non-biological) concomitants, such as electrode positioning, effective stimulus strength, depth in slice, etc. These considerations make the comparison of such responses between different astrocyte recordings largely uninterpretable.

We next focused on the relationship between Kir4.1 expression and the kinetics of astrocytic K^+^ currents induced by synaptic activity. In response to a brief electrical stimulation of Schaffer collaterals (5 stimuli × 50 Hz), Kir4.1* astrocytes displayed significantly faster K^+^ kinetics than control astrocytes (decay time 1.13 ± 0.15 and 1.85 ± 0.09 s, *n =* 11 and *n* = 15, respectively; Figure 2D), suggesting an accelerated removal of the excess [K^+^]_out_ by the former cells. Because the Kir4.1-dependent control of K^+^ homeostasis is critical for the efficient electrogenic glutamate uptake by astrocytes,^12,17,57^we next compared the synaptically evoked glutamate uptake current between control and Kir4.1* astrocytes. The current (*I*_*GluT*_) was isolated using the glutamate transporter blocker TBOA (50 μM), as described earlier.^6^ We found that the progressive use-dependent elevation of the uptake current was drastically diminished in Kir4.1* astrocytes (Figures 2E and 2F), which was a consequence of a much faster current decay kinetics in these cells compared with control astrocytes (Figure 2F; decay time 26.1 ± 2.8 and 12.9 ± 2.7 ms, *n* = 11 and *n* = 6, respectively; *p* < 0.01, *t* test). Again, the absolute amplitude of astrocyte currents recorded in these experiments varied widely depending on each particular experimental setting (stimulating electrode position with respect to the patched cell, depth in slice, etc.; Figure S3), thus compelling us to focus on the time-domain measures, such as decay times. Overall, the above observations suggest that the upregulation of astrocytic Kir4.1 could speed up K^+^ removal from the extracellular space, thus also accelerating use-dependent glutamate uptake.

### The expression level of Kir4.1 territorially regulates Ca^2+^ homeostasis in presynaptic axons

**Figure 3:**
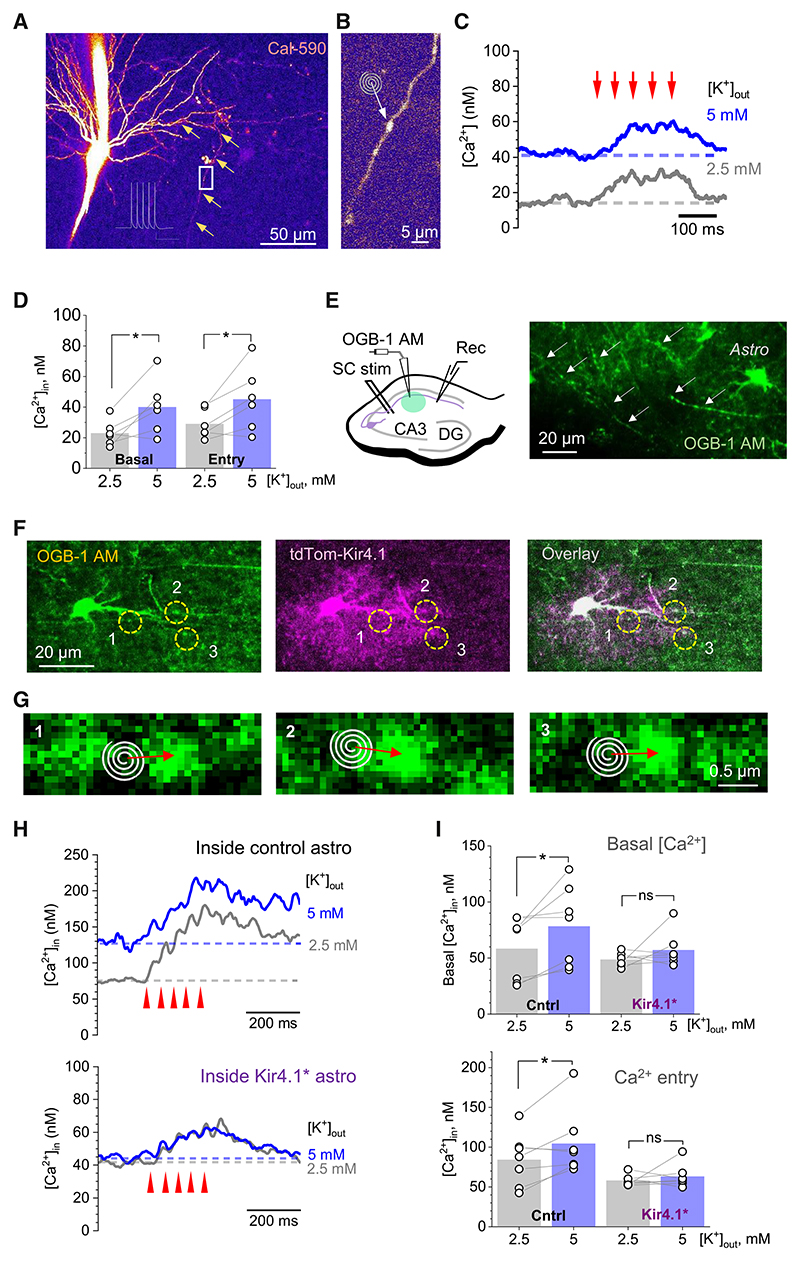
High K^+^-driven increases in pre-synaptic Ca^2+^ do not occur within Kir4.1* astrocyte territories (A) A pyramidal neuron dialyzed whole cell with 300 μM Cal-590; inset, soma stimulation protocol, five action potentials at 20 Hz; arrows, tracked axon; rectangle, region of interest; λ_x_^2P^ = 910 nm. Scale bar, 50 μm. (B) Region of interest (rectangle in A) magnified, focusing on the targeted presynaptic bouton; spiral + arrow, the “Tornado” (spiral) linescan position over the axonal bouton. Scale bar, 5 μm. (C) Cal-590 FLIM-calibrated presynaptic [Ca^2+^] dynamics at the bouton shown in (B), under 2.5 mM (gray) and 5 mM (blue) of [K^+^]_out_, as indicated; arrows, Schaffer collaterals stimuli (5 × 20 Hz). (D) Summary of experiments in (C) (mean + data points), for the baseline presynaptic [Ca^2+^] and Ca^2+^ entry ([Ca^2+^] increment), as indicated; dots, readouts from individual boutons; *p < 0.05 (*n* = 6, paired *t* test). (E) Schematic, experimental arrangement; image panel, characteristic examples of individual tracked Schaffer collateral axons (arrows) filled with OGB-1 AM; λ_x_^2P^ = 800 nm. Scale bar, 20 μm. (F) Multiplexed imaging of OGB-1 filled Schaffer collateral axons (green channel) that trespass a Kir4.1* astrocyte territory (magenta channel); circles (1, 2, 3), axonal boutons of interest. Scale bar, 20 μm (applied throughout). (G) Axonal boutons shown in (F) magnified, with Tornado linescan positions for imaging, as indicated. Scale bar, 0.5 μm (applied throughout). (H) Characteristic examples of the FLIM-calibrated presynaptic [Ca^2+^] dynamics recorded at an axonal bouton under 2.5 and 5 mM of [K^+^]_out_, either inside Kir4.1* astrocyte territories or outside (control), as indicated; arrows, Schaffer collaterals stimuli (5 × 20 Hz). (I) Summary of experiments in (H) (mean + data points), for the basal presynaptic [Ca^2+^] and [Ca^2+^] Ca^2+^ entry ([Ca^2+^] increment), as indicated; dots, readouts from individual boutons; *p < 0.05 (*n* = 7 in both cases, paired *t* test).

Because activity-induced [K^+^]_out_ elevations would depolarize neuronal membranes, the latter could result in the widening of the axonal action potentials, thus leading to an increase in presynaptic Ca^2+^ entry.^58^ The potentially important role of Kir4.1 in the use-dependent plasticity of excitatory transmission has already been suggested.^23^ Therefore, we first examined the effect of elevated [K^+^]_out_ on presynaptic Ca^2+^ at individual axonal boutons traced from the CA3 pyramidal cell soma held in whole cells (Figures 3A and 3B). Cell dialysis with the red-shifted Ca^2+^ indicator Cal-590, which was FLIM calibrated for [Ca^2+^] at the same setup previously,^59^ enabled FLIM-aided monitoring of axonal [Ca^2+^] in the nanomolar range (Figure 3C). On average, elevating [K^+^]_out_ from 2.5 to 5 mM raised both basal [Ca^2+^] and spike-evoked peak presynaptic Ca^2+^ entry by 74% and 55%, respectively (Figure 3D).

The success rate of tracing the axon of a patched cell, as illustrated above, into the territory of a single selected astrocyte is extremely low. We therefore modified the above experiment to achieve workable sampling. First, we bolus-loaded a small stratum radiatum area with the membrane-permeable Ca^2+^ indicator OGB-1 AM, allowing us to trace, away from the area, individual Schaffer collaterals against a relatively unstained background (Figure 3E). Second, we could identify OGB-1-filled axonal fragments that trespass the territories of TdTom-labeled, either control or Kir4.1* astrocytes (Figure 3F; the astrocytic soma and large branches could also pick up OGB-1 staining). Finally, we monitored [Ca^2+^] in individual axonal boutons filled with OGB-1, inside the TdTom-labeled territories (Figure 3G), using highly sensitive FLIM readout (as in Figures 2B or 3A). We found that, in control astrocytes, increasing [K^+^]_out_ from 2.5 to 5 mM elevated both presynaptic basal [Ca^2+^] and spike-evoked peak Ca^2+^ entry, similar to single-cell experiments (compare Figures 3H, top, and 3C: comparatively higher basal [Ca^2+^] in the former case is expected due to the residual uptake of OGB-1 AM by intracellular organelles). However, inside the territories of Kir4.1* astrocytes, the influences of [K^+^]_out_ elevations were effectively eliminated (Figures 3H, bottom and 3I). Although it is difficult to assess the exact relationship between bathapplied [K^+^]_out_ and exact [K^+^]_out_ near the recording site, the most parsimonious explanation of these results is that individual Kir4.1* astrocytes are more rapid and/or more efficient in achieving the equilibrated [K^+^]_out_ levels inside their territories than their control counterparts.

### The expression level of Kir4.1 territorially regulates evoked glutamate release and synaptic plasticity

**Figure 4:**
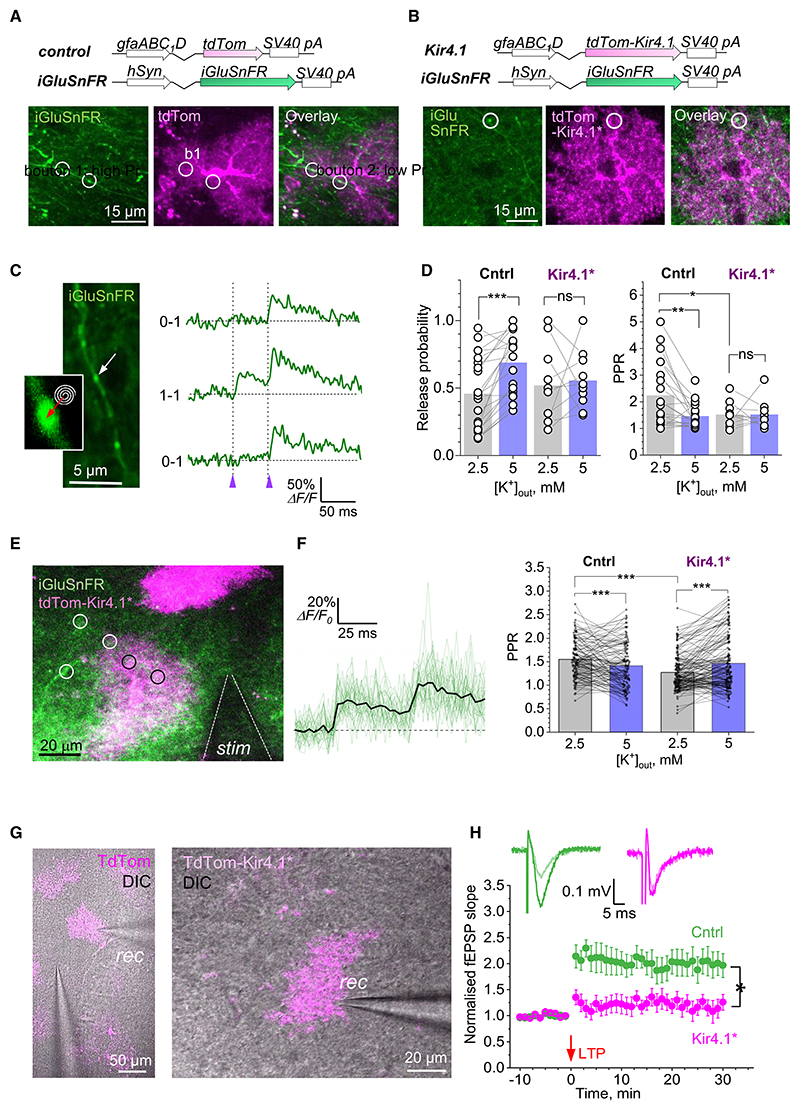
High K^+^-driven increases in synaptic release probability do not occur within Kir4.1* astrocyte territories (A) Diagram, AAV vectors for TdTom (gfaABC1D promoter, control) and iGluSnFR (hSyn promoter). Image panels: multiplexed imaging of iGluSnFR-expressing Schaffer collaterals (green channel) trespassing the territory of a control labeled astrocyte (red channel, shown as magenta), as indicated. Scale bar, 15 μm. (B) Diagram: AAV vectors for TdTom-Kir4.1 (gfaABC1D promoter, control) and iGluSnFR (hSyn promoter). Image panels: multiplexed imaging of iGluSnFR-expressing Schaffer collaterals (green channel) trespassing the territory of a Kir4.1* astrocyte (magenta), as indicated. Scale bar, 15 μm. (C) Image: example of a traced iGluSnFR-expressing axonal bouton (arrow); inset: the bouton image magnified, with the Tornado linescan position shown. Traces: examples of iGluSnFR-reported single-bouton quantal glutamate responses (successes or failures) to paired-pulse stimuli at 20 Hz. Scale bar, 5 μm. (D) Summary of experiments in (C) (mean + data points) reporting release probability (left) and paired-pulse ratios (PPRs, right) calculated based on quantal release scores, under 2.5 and 5 mM of [K^+^]_out_, inside of either control or Kir4.1* astrocyte territories (*n* = 21 and 10, respectively), as indicated; mean ± SEM, dots, readouts from individual synapses; **p < 0.01, ***p < 0.005 (paired *t* test). (E) Multiplexed imaging (green iGluSnFR and red TdTom channels) of a stratum radiatum area depicting the stimulating electrode (*stim*), and examples of ROIs focused on axonal boutons that fall inside (black circles) or outside (white circles) of TdTom-labeled Kir4.1* astrocytes. Scale bar, 20 μm. (F) Traces: a typical example of the ROI-delimited glutamate-sensitive iGluSnFR response to paired-pulse stimulation (green, 31 individual traces; black, average). Graphs: summary (mean + data points) of the PPR values, under 2.5 and 5 mM of [K^+^]_out_, either outside (control) or inside the TdTom-Kir4.1* astrocyte territories (*n* = 112 and 146 ROIs, respectively), as indicated; dots, individual ROI readouts; ***p < 0.005 (paired *t* test). (G) Examples of the recording pipette positioning (*rec*) for documenting fEPSPs within the territory of control (TdTom, left) and TdTom-Kir4.1* (right) astrocytes; DIC + TdTom (magenta) channels combined. Scale bars, 50 μm (left) and 20 μm (right). (H) Traces: examples of fEPSPs recorded before (lighter) and after (darker) LTP induction protocol, recorded inside control (TdTom, left) and TdTom-Kir4.1* (right) astrocyte territories. Graph: time course of the fEPSP slope (normalized to baseline, mean ± SEM) recorded inside the control (*n* = 10) and TdTom-Kir4.1* (*n* = 8) astrocytes, as indicated; arrow, LTP induction protocol; dots, individual slice data; *p < 0.05 (*t* test for the means over 30–32 min period post-induction).

Since the dynamics of presynaptic Ca^2+^ control spike-evoked release of the excitatory neurotransmitter glutamate, we sought to monitor glutamate release from individual presynaptic boutons in conditions that replicate the above experiments. To this end, we first expressed the optical glutamate sensor iGluSnFR (AAV9.hSynap.iGluSnFR.WPRE.SV40) in area CA1 (methods). Next, we focused on the iGluSnFR-labeled axons (Schaffer collaterals) trespassing the territories of the TdTom-labeled, either control or Kir4.1* astrocytes (Figures 4A and 4B). Applying a minimal stimulation protocol revealed axonal boutons that responded to paired stimuli (50 ms apart) with glutamate release successes or failures (Figure 4C). This approach enabled us to directly calculate the average release probability P_r_ and paired-pulse ratio (PPR) at the monitored presynaptic boutons, as described previously.^59,60^ Firstly, we found that in baseline conditions (2.5 mM [K^+^]_out_) Kir4.1 overexpression had no detectable effect on P_r_ of local synapses (Figure 4D, left, gray bars). In similar conditions, however, the PPR was significantly smaller inside Kir4.1* areas than in the control (Figure 4D, right, gray bars). What could be the mechanism underlying this apparent contradiction? Within the territories of control astrocytes, elevating [K^+^]_out_ to 5 mM increased P_r_ substantially (from 0.46 ± 0.06 to 0.69 ± 0.05, *n =* 21, *p* < 0.001, *t* test), indicating that P_r_ could increase or decrease with the corresponding changes in [K^+^]_out_. We have also shown that, during synaptic activity, Kir4.1* astrocytes return [K^+^]_out_ to their resting concentration faster than control astrocytes (Figure 2D). The latter suggests that the [K^+^]_out_ elevation following a synaptic discharge should be reduced or shortened within Kir4.1* compared with control territories, thus leading to lower P_r_ for the immediate second discharge, and hence reduced PPR, at local synapses. Thus, basal synaptic transmission is not affected by Kir4.1 overexpression, whereas short-term plasticity is. Consistent with these mechanisms, we also found that, inside the Kir4.1* astrocyte territories, the increase in [K^+^]_out_ to 5 mM had little effect on either P_r_ or PPR (Figure 4D, Kir4.1*). These results also suggest that PPR is not always a reliable indicator of changes in P_r_ under varied conditions of use-dependent axonal excitability.

To extend our sample to a broader population of randomly sampled synapses, we modified the experiment so that we could compare paired-pulse evoked iGluSnFR responses between the groups of synapses outside (hence control) and inside Kir4.1* astrocyte territories in multiple places (Figure 4E) using a resonant scanner (see methods). In these tests, Schaffer collateral evoked fluorescent responses normally displayed no failures (Figure 4F, traces), reflecting glutamate released and “spilled over” from more than one neighboring synapse, as reported previously.^48,61^ While a direct evaluation of P_r_ in these conditions was not feasible, the reduction in PPR in control areas upon [K^+^]_out_ elevation, and in Kir4.1* compared with control territories under 2.5 mM [K^+^]_out_ (Figure 4F, bar graphs), was fully consistent with our single-synapse data (Figure 4D). Here, the “overshoot” in PPR readout within Kir4.1* territories under 5 mM [K^+^]_out_ (Figure 4D) could be due to a complex relationship between the altered K^+^ current kinetics, glutamate spillover, and axonal excitability upon [K^+^]_out_ elevation in conditions of multiple afferent stimulation, which might require a separate investigation. Again, the reduced PPR within Kir4.1* territories (compared with control ones) under 2.5 mM [K^+^]_out_ suggests an accelerated [K^+^]_out_ re-equilibration by Kir4.1* astrocytes after the first synaptic discharge.

Earlier reports associated inhibited Kir4.1 expression with substantially enhanced synaptic potentiation.^23,31^ We therefore asked if the overexpression of Kir4.1 would affect the induction of synaptic long-term potentiation (LTP) at the CA3-CA1 synaptic circuit.^62^ Strikingly, the classical high-frequency stimulation protocol that had sufficient stimulation strength to induce LTP inside the control (TdTom-labeled) astrocytic areas, failed to do so inside the Kir4.1* astrocyte territories (Figures 4G and 4H). This “yes-no” outcome points to the failure of induction, in the present settings. Based on our observations outlined above, the most parsimonious explanation for the latter must be the accelerated removal of local extracellular K^+^ and glutamate by Kir4.1* astrocytes during high-frequency stimulation. This could correspond to the overall reduced pre- and postsynaptic excitability, including lowered P_r_, during the induction protocol, thus preventing critical depolarization of the postsynaptic neuronal dendrites within the Kir4.1* territories.

### Kir4.1* astrocytes attenuate K^+^ waves of cortical spreading depolarization

**Figure 5:**
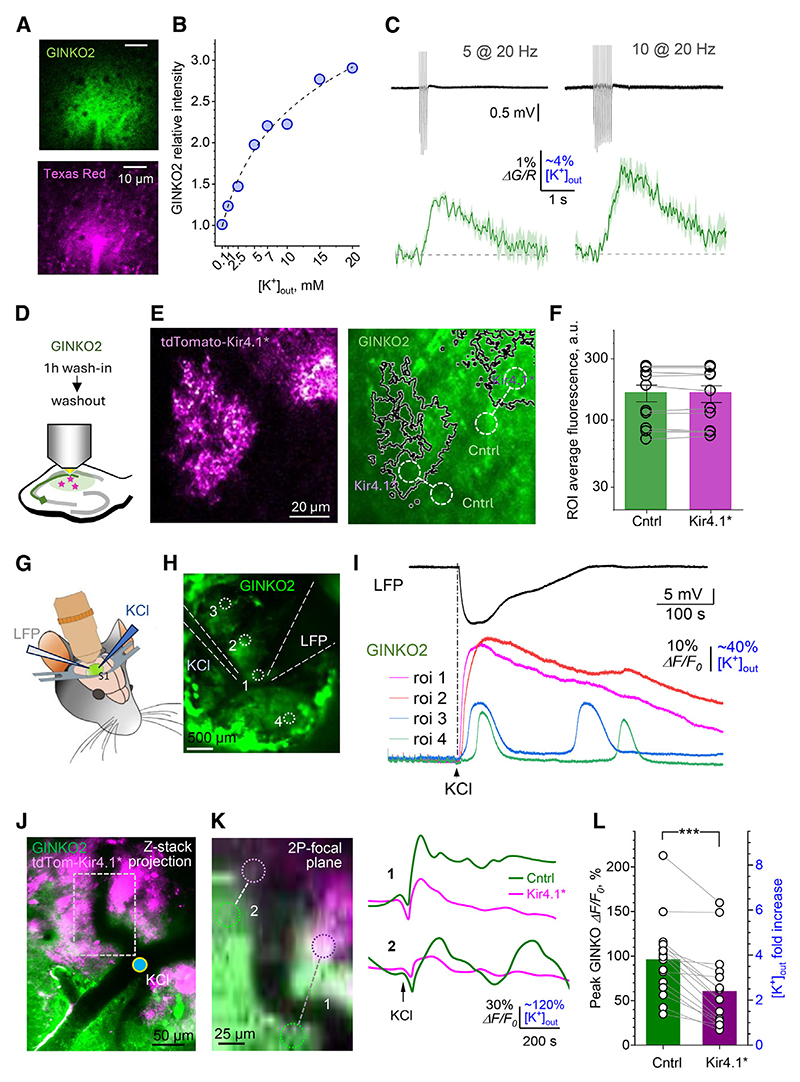
Kir4.1* astrocytes can locally attenuate [K^+^]_out_ elevation during cortical spreading depression *in vivo* (A) Experimental arrangement for ratiometric measurement of [K^+^]_out_ using the extracellular [K^+^]-sensitive sensor GINKO2 and [K^+^]-insensitive Texas red ejected from a pressurized pipette, shown in green and red emission channels, as indicated (λ_x_^2P^ = 940 nm; depth in slice, 50–60 μ m). Scale bar, 10 μm. (B) Summary of the calibration experiment shown in (A): relative GINKO2 fluorescence intensity (green/red); dotted line, logarithmic approximation *y* = 9340 ln(*x* + 2:74). (C) Examples of an optical response generated by extracellular GINKO2 to 5 (left) and 10 (right) electrical stimuli at 20 Hz applied to Schaffer collaterals; mean ± SEM (shaded area), *n* = 6 trials. (D) Diagram, experimental arrangement for imaging extracellular GINKO2 (see methods). (E) Examples of multiplexed imaging depicting Kir4.1* astrocytes (red channel, magenta) and immobile extracellular GINKO2 (green channels) in the same tissue area; connected circles, paired ROIs for sampling GINKO2 intensity values inside and outside (Cntrl) Kir4.1* territories (delineated by black lines), as indicated. Scale bar, 20 μm. (F) Summary (bars, mean ± SEM) of the GINKO2 fluorescence intensity values sampled inside and outside (Cntrl) Kir4.1* territories, as indicated; dots, individual astrocyte territory ROI data; connecting lines show paired values as in (F) (*n =* 10 astrocyte territories). (G) Diagram, experimental arrangement for the high K^+^-induced cortical spreading depolarization. (H) A view through the cranial window in an anesthetized mouse (green channel, extracellular GINKO2; bath application 1 h before imaging; λ_x_^1P^ = 470 nm); the micropipettes for local field potentials (LFP) recording and for KCl application are shown, as indicated; 1–4 circles, ROIs for fluorescence recording. Scale bar, 500 μm. (I) Top: LFP trace recorded in experiment shown in (H) during high KCl application (arrow); bottom: GINKO2 *ΔF/F*_*0*_ signal traces recorded simultaneously in ROIs shown in (H). (J) Cortex view through the cranial window, with a multiplexed imaging overlay (GINKO2 channel and TdTomato-Kir4.1* astrocyte channel) depicting Kir4.1* astrocyte areas (magenta) and an approximate point of KCl application (circle); blood vessels (dark areas) can be seen crossing the view; averaged 50 μm z stack; λ_x_^2P^ = 940 nm. Scale bar, 50 μm. (K) Image, fragment of the view in (J) magnified and shown in a single 2PE focal plane; connected circles 1–2, two pairs of ROIs (characteristic example) reporting GINKO2 fluorescence inside and outside the visible Kir4.1* astrocyte territories. Traces: GINKO2 *ΔF/F*_*0*_ signals recorded at the ROI pairs 1–2 during high KCl application, as indicated. Scale bar, 25 μm. (L) Summary graph (mean + sampled data points) of experiments in (G); ***p < 0.001 (*n =* 17 ROI pairs; paired *t* test).

Significant [K^+^]_out_ elevations in the brain have long been attributed to abnormal network behaviors, such as seizures and cortical spreading depolarization (CSD), which is characterized by waves of [K^+^]_out_-driven depolarization.^4,8,63^ However, our understanding of the dynamic [K^+^]_out_ landscapes during CSD has been limited to single (or few)-point recordings by K^+^-sensitive electrodes. We therefore first sought to obtain the readout of area-limited [K^+^]_out_ changes by loading the extracellular space with the recently developed (green) fluorescent K^+^ sensor GINKO2^64^ and calibrating it for [K^+^]_out_. Because bath application of the K^+^-binding sensor appeared to lead to poorly controlled K^+^ buffering throughout the tissue, we first locally applied GINKO2 and the soluble red fluorescent indicator Texas red via a micropipette (Figure 5A) to obtain a ratiometric reading (green/red) of the K^+^-dependent fluorescent signal at different [K^+^]_out_. The resulting dependence indicated a ~200% change in GINKO2 fluorescence over the [K^+^]_out_ change from micromolar levels to ~10 mM (Figure 5B). We next used this approach to evaluate average [K^+^]_out_ elevations in response to mild synaptic activity. Five or 10 electrical stimuli (at 20 Hz) applied to Schaffer collaterals, generated readily detectable fluorescence signals that corresponded to [K^+^]_out_ increases of ~0.05 or ~0.1 mM, respectively (Figure 5C). This range appears in good correspondence with the theoretical estimate outlined in the introduction above, with the readout of K^+^-sensitive electrodes,^30^ and with the classical [K^+^]_out_ measurements relating 5–10 μM [K^+^]_out_ elevations to a sequence of several hundred of synchronous afferent discharges.^1^

Equipped with the [K^+^]_out_ optical measuring method, we first asked whether Kir4.1* maintain [K^+^]_out_ levels that are different from those within control (wild-type [WT]) territories. To avoid having GINKO2 as a diffusible extracellular K^+^ buffer, we first bath applied and then washed out the dye protein leaving its significant fraction immobile in the extracellular space (Figure 5D), which should provide sufficient K^+^ sensitivity^64^ (also see below). We could thus compare quantitatively average GINKO2 fluorescence within and outside Kir4.1* territories across multiple astrocytes in hippocampal slices (Figure 5E). The outcome suggested no detectable difference in [K^+^]_out_ (Figure 5F). This is consistent with a biophysical prediction that a change in the Kir4.1 channel density should not affect the transmembrane K^+^ concentration gradient because active K^+^ transport systems, such as Na^+^/K^+^-ATPase, should restore the equilibrium.

Finally, we asked whether the overexpression of Kir4.1 would affect the dynamics of [K^+^]_out_ landscapes during CSD. Because extracellular bolus-loading GINKO2 *in vivo* appears to maintain its K^+^ sensitivity,^64^ we applied the sensor to the cortical surface under the experimental protocol of high-potassium-induced CSD as reported earlier^65^ (Figures 5G and 5H). Monitoring both K^+^-dependent GINKO2 fluorescence and local field potential in control experiments, we confirmed that the protocol induced a clear depolarization wave accompanied by both increases and self-sustained [K^+^]_out_ waves peaking at the level severalfold higher than the resting [K^+^]_out_ (Figure 5I), as expected.^3,4,8^

We next reproduced this protocol in the animals overexpressing Kir4.1 in TdTom-labeled cortical astrocytes. First, we found a cortical region with clearly identifiable Kir4.1* astrocytes and placed the high-potassium micropipette in the vicinity (Figure 5J). Next, to exclude signal contamination, we focused on a single focal plane (in two-photon excitation mode) and monitored K^+^-sensitive GINKO2 fluorescence at paired regions of interest of a similar size, one inside the Kir4.1* astrocyte territory, and one outside nearby (Figure 5K, image). Once the high-potassium pulse was delivered to induce CSD, the paired GINKO2 recordings were documented for at least several minutes afterward (Figure 5K, traces). In all recorded paired locations (*n =* 16) the waves of [K^+^]_out_ were clearly attenuated within the Kir4.1* astrocyte territory compared with the area outside (Figure 5L).

### Handling [K^+^]_out_ by Kir4.1* astrocytes: Biophysical underpinning

**Figure 6:**
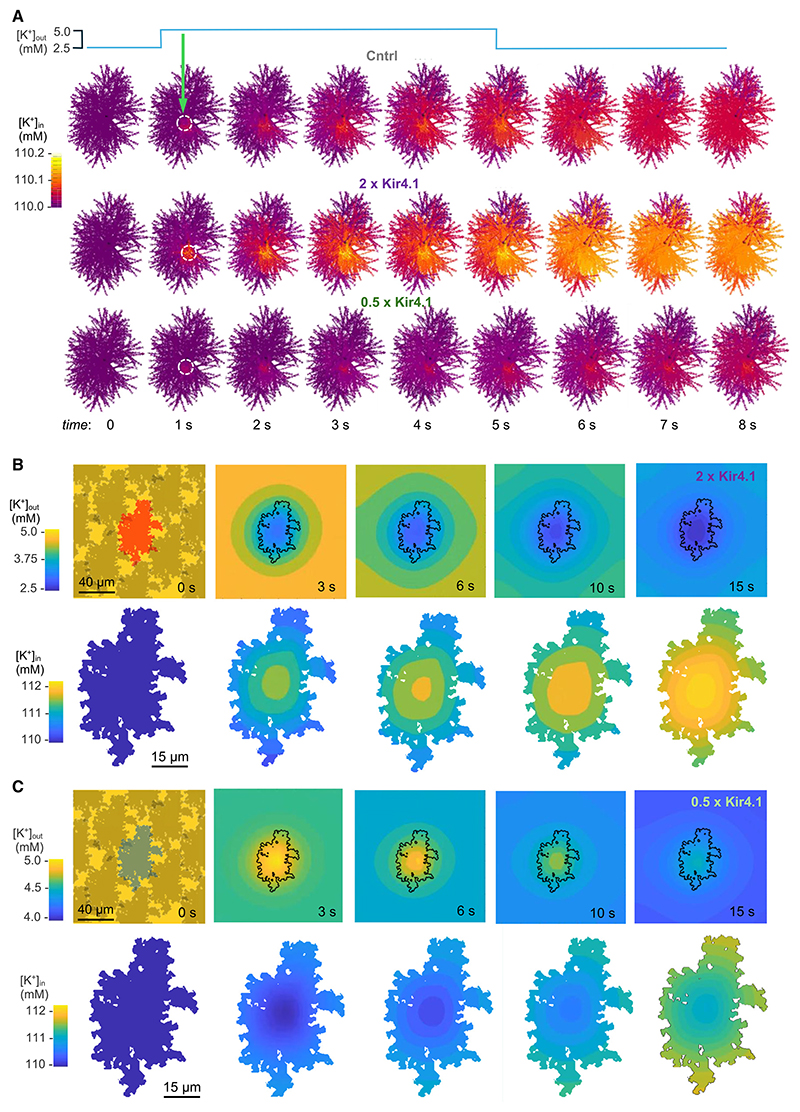
Overexpression of Kir4.1 in a single astrocyte boosts local sink of [K^+^]_out_: biophysical evaluation (A) Simulated dynamics of [K^+^]_in_ in a 3D realistic CA1 astrocyte model^26^ (see methods), following a 5 s long elevation of [K^+^]_out_ from 2.5 to 5 mM (top trace) within a 10 mm sphere near the astrocyte center (dashed circle), with the Kir4.1 expression at the control (top row), doubled (middle), and halved (bottom) density, as indicated. Simulations performed using the modeling platform BRAINCELL (www.neuroalgebra.net), a multi-functional expansion of NEURON.^67^ Note small (<1%) changes in [K^+^]_in_. See Figure S5 for the simulated dynamics of [K^+^]_out_ in similar settings. (B) Simulated 3D dynamics (shown in central section) of [K^+^]_out_ outside and [K^+^]_in_ inside a modeled CA1 astrocyte^26^ that overexpresses Kir4.1 2-fold, is positioned in the center, and surrounded by control astrocytes; the latter are represented by their Kir4.1 channel kinetics and density (see methods); note varied color scale bars. Scale bars, 40 μm (top row) and 15 μm (bottom row). (C) Simulations settings as in (B), but for the halved expression of Kir4.1 for the central astrocyte; other notations as in (B).

Our observations of Kir4.1 actions, combined with previously reported experimental data on CA1 astrocyte morphology and physiology^23,26,34,66^enable us to examine and possibly understand the underlying biophysics using detailed computational modeling. In the first test, we explored how a local increase in [K^+^]_out_ redistributes the intracellular K^+^ concentration ([K^+^]_in_) inside astrocytes that express varied levels of Kir4.1. For the detailed cell model, we adopted the fine architecture, membrane mechanisms including Kir4.1, and intra- and extracellular environment features from one of the 3D-reconstructed CA1 astrocytes with regenerated nanoscopic features, as reported previously.^26^ Here, the control astrocyte had the previously established Kir4.1 conductance density of 0.175 mS/cm^2^, whereas the test astrocytes had the conductance either doubled, at 0.35 mS/cm^2^ (to correspond to the respective difference in K^+^ current; Figures 1C and 1D), or halved, for comparison purposes. The latter scenario reflects the fact that a loss or reduced expression of Kir4.1 has long been associated with a variety of neurological conditions.^36–43^ We next elevated [K^+^]_out_ from its resting level of 2.5–5 mM within a 10-mm-wide spherical volume near the center of the 3D astrocyte territory (Figure 6A), to mimic several hundred of spikes occurring within a group of ~1,000 synapses (at a synaptic density in CA1 area of ~2 μm^−3^) for 5 s, in line with our measurements of activity-dependent [K^+^]_out_ rises. We then monitored the dynamic 3D landscape of [K^+^]_in_ inside the modeled astrocytes. Because the resting [K^+^]_in_ in astrocytes is in the range of ~110 mM, whereas the local [K^+^]_out_ change was only 2.5 mM, it was no surprise that the [K^+^]_in_ redistribution involved <1% variations in the concentration level, in both control and test cases (Figure 6A). Such negligible changes suggest no need for powerful mechanisms of K^+^ extrusion or spatial siphoning from astrocytes during mild synaptic activity. In a “complementary” test, we sought to assess how, in similar settings, the varied Kir4.1 expression affects the rate at which a brief local increase in [K^+^]_out_ decays outside the astrocyte in question. The results suggest that the speed of [K^+^]_out_ equilibration post-elevation scales roughly with the astrocyte Kir4.1 expression level (Figure S6).

Finally, we asked to what comparative degree an individual astrocyte that over- or underexpresses Kir4.1 and is surrounded by control (WT) astrocytes handles extracellular K^+^ elevations while dissipating it inside the cell. We therefore employed our BRAINCELL simulation platform (www.neuroalgebra.net) to place our realistic 3D astrocyte model^26^ in the center of a 3D neuropil that is evenly filled with the K^+^ uptake mechanisms that represent astrocyte Kir4.1 action (as in Figure 6A) in control conditions (see methods). Our simulations could thus derive the spatiotemporal dynamics of extracellular and intra-astrocyte K^+^ after a brief elevation of [K^+^]_out_ from 2.5 to 5 mM when the “central” astrocyte expresses, for instance, either a doubled (Figure 6B) or halved (Figure 6C) Kir4.1 level compared with the WT control. The outcome provides a quantitative illustration of how the varied level of Kir4.1 expression in an individual astrocyte could affect the local dynamic landscapes of [K^+^]_in_ and [K^+^]_out_ during neuronal network activity (Figures 6B and 6C).

## Discussion

### Small volume-average K^+^ elevations may mask K^+^ hotspots

Classical observations have related intense neuronal activity (hundreds of highly synchronized discharges over several seconds or minutes) to the [K^+^]_out_ elevations in the several-millimolar range.^1,4^ Such experiments revealed the roles of NKCC1 exchanger and NKA pumps in removing large excesses of [K^+^]_out_.^13–15^ However, short bursts of afferent activity prompted astrocytic K^+^ currents that should correspond to the local volume-average [K^+^]_out_ rises of only several percent.^6,23,30^ Here, we used the novel K^+^ sensor GINKO2 to confirm this (Figures 5A–5C). In fact, a similar estimate arises from the readout of K^+^-sensitive electrodes,^30^ or assuming near-linear summation of [K^+^]_out_ elevations during long-train stimulation.^1^

In such moderate conditions, the bulk of astrocyte K^+^ current was found to flow through Kir4.1 channels,^23^ consistent with observations that associate increased NKA activity with membrane depolarization^19,20^ expected during substantial increases in [K^+^]_out_. However, increases of [K^+^]_out_ averaged over a tissue volume do not provide details about the possible (microscopic) [K^+^]_out_ hotspots at the sites of concentrated synaptic activity, especially at the sites of K^+^ efflux through axonal K^+^ channels^47^ or through postsynaptic NMDA receptors that may remain activated for 100–200 ms.^6^ Such activity-driven hotspots have been suggested to prompt local NKCC1-dependent morphological plasticity of astroglia, leading to a change in local rules of signal integration.^5^ It would seem important to understand the prevalence and distribution of such K^+^ hotspots in the future, which has been difficult to ascertain with the current version of GINKO2.

### Overexpression of Kir4.1 speeds up K^+^ and glutamate uptake

Increasing the number of astrocyte Kir4.1 channels, in the present conditions, leads to slight cell hyperpolarization and a drop in cell input resistance (Figure 1B). These phenomena are consistent with the increased influence of the negative K^+^ equilibrium potential under an increased overall conductance of the inwardly rectifying Kir4.1 channels. Indeed, Kir4.1 overexpression led to a 2-fold increase in K^+^ conductance (Figures 1C and 1D). Firstly, this accelerated K^+^ uptake, and hence the removal of [K^+^]_out_ excess (Figure 2D). Secondly, because ([K^+^]_out_-dependent) astrocyte depolarization reduces the activity of glutamate transporters^68^ that are responsible for the bulk of glutamate buffering in the brain, the increased rate of extracellular K^+^ removal (hence the increased rate of repolarization) produces a substantially accelerated glutamate uptake, particularly prominent under repetitive activity (Figure 2E). These phenomena must reduce local neuronal excitability and limit activity-driven extrasynaptic glutamate escape,^6,69^ but also lead to a change in synaptic fidelity and its use-dependent plasticity, as briefly discussed below. Intriguingly, Kir4.1 expression in astrocytes varies during development and among brain (sub)regions,^70^ suggesting that such variability could be one of the mechanisms regulating local excitatory activity.

Interestingly, Kir4.1 overexpression alters neither the basal Ca^2+^ level nor its spontaneous elevations if compared with control astrocytes (Figures 2A–2C, S1, and S2). Whether other modes of (spontaneous or evoked) astrocytic Ca^2+^ activity are affected by Kir4.1 overexpression, *in situ* or in the intact brain, remains an important and intriguing question. Inducing an evoked Ca^2+^ response in an astrocyte requires strong stimulation of multiple afferents, which involves a multitude of poorly controlled experimental concomitants, such as stimulating electrode positioning, depth in slice, stimulus strength, etc., thus making it difficult to compare reliably the readouts of individual experiments in this context.

### Astrocytes exert territory-delimited control of synaptic fidelity

In baseline conditions under 2.5 mM [K^+^]_out_, we did not detect significant differences between the territories of control and Kir4.1* astrocytes in axonal presynaptic basal [Ca^2+^], action potential evoked Ca^2+^ entry (Figure 3I), or quantal release probability (Figure 4D) displayed by the trespassing axons. However, the synapses trespassing Kir4.1* territories showed a reduced PPR, indicating a significant change in short-term plasticity rules (Figures 4D and 4F). As detailed in the results, this change could be explained by the accelerated removal of [K^+^]_out_, and hence reduced release probability, over a short period of time after the first synaptic discharge.

Another conspicuous effect was observed when [K^+^]_out_ in the bath medium was raised to 5 mM. This led to an increase in presynaptic basal [Ca^2+^], evoked Ca^2+^ entry, and release probability at synapses within the territories of control astrocytes (Figures 3D, 3I, 4D, and 4F). Such increases reflected the well-established relationship between increased [K^+^]_out_, local membrane depolarization, and increased spontaneous and spike-evoked opening of voltage-gated presynaptic Ca^2+^ channels.^71,72^ In contrast, synaptic fidelity inside Kir4.1* astrocyte territories remained unaffected (Figures 3I and 4D), indicating a strong influence of Kir4.1 overex-pression on the astrocyte territory-delimited K^+^ homeostasis and its physiological consequences.

In theory, further potentially interesting insights into the adaptive roles of Kir4.1 could be obtained by reducing Kir4.1 presence, using either non-saturating concentrations of its specific blocker VU0134992^50^ or an RNA-silencing technique that we used previously.^73^ However, degrading the Kir4.1 function would take us into the realm of pathological changes in neural function, as demonstrated in multiple studies,^36–39,42,43^ whereas overex-pressing Kir4.1 prompts changes that could be interpreted as physiological and potentially relevant for therapeutic targeting. We have therefore limited our exploration to the latter. At the same time, our detailed biophysical simulations illustrate quantitatively the role of Kir4.1 expression level, below or above the control, in shaping the 3D dynamic landscape of [K^+^]_out_ and [K^+^]_in_ in response to transient elevations of [K^+^]_out_ (Figure 6).

### Sparsely distributed Kir4.1* astrocytes weaken spreading depolarization

The phenomenon of CSD^8^ is associated with a number of neurological disorders including migraine with aura, traumatic brain injury, stroke, glioblastoma, and epilepsy.^74^ Given its importance, a recent resurgence in the new approaches and tools aimed at its exploration has been reported.^63,65^ That the sparsely distributed Kir4.1* astrocytes can locally attenuate the spreading waves of high extracellular K^+^ upon CSD initiation (Figures 5G–5L) brings about two conclusions. Firstly, in the intact brain, individual Kir4.1 overexpressing astrocytes can maintain within their territories altered K^+^ homeostasis. Secondly, an accumulation of Kir4.1* astrocytes in the brain areas associated with the focal origin of spreading depolarization could represent an important means of reducing the latter. Overall, our findings unveil some fundamental relationships between astrocytic Kir4.1 expression, its territorial effect on local K^+^ homeostasis, and its consequences for excitatory transmission. Whether Kir4.1 overexpression in astrocyte populations could have a significant effect on the initiation or prevalence of seizures, and to what degree it interferes with cognitive functions of the brain, will be an important quest to pursue.

### Individual astrocytes can buffer significant extracellular K^+^ rises

The paradigm of K^+^ buffering involving diffusion across the gap junction-interconnected astrocyte syncytium has provided an elegant explanation for the mechanism of maintaining K^+^ homeostasis in the brain.^12,75,76^ However, experiments with genetic manipulations of gap junction proteins have questioned the necessity of inter-astrocyte K^+^ diffusion for the successful removal of [K^+^]_out_ excess under physiological neuronal firing,^30,77^ even though astrocyte interconnectivity appears important during excessive excitation such as seizures^30,78^ and for regulating [K^+^]_out_ during afferent burst activity.^79^

Indeed, astrocytes maintain [K^+^]_in_ in the region of 100–115 mM, whereas physiological brain activity is unlikely to elevate [K^+^]_out_ by more than 2–2.5 mM for limited time periods. With the extracellular/intra-astrocyte volume ratio of approximately 2,^25,26^ the influx of 2–2.5 mM [K^+^]_out_ would represent a 4%–5% change in [K^+^]_in_. Such a change would cause only a tiny K^+^ efflux current when [K^+^]_out_ is back to its resting level,^34^ and there is no reason to believe it would have a detectable effect on astrocyte physiology. Further still, moderate neural activity is likely to produce only hotspots of [K^+^]_out_ elevations, and detailed modeling of realistic 3D astrocyte morphology^26^ suggests that, in such cases, the redistribution (spatial buffering) of internal K^+^ involves [K^+^]_in_ changes on the <1% scale (Figure 6A). These data propose that, under “normal” physiological activity, individual astrocytes are capable to cope with local rises of extracellular K^+^ induced by neuronal firing.

### Limitations of the study

This study relies significantly on experiments conducted with acute brain slices. While this preparation has led to numerous universally recognized discoveries over decades, it also has notable limitations compared with the intact brain. The trauma of slicing, disrupted connections, and the constraints of slice life support systems may introduce poorly understood consequences for brain cell physiology in specific experimental designs. Consequently, caution is warranted when directly extrapolating data from brain slices to the intact brain. This consideration prompted us to validate our key conclusions through *in vivo* experiments (Figure 5). Another conceptual limitation is that, for feasibility reasons, we tested only a limited set of basic regimes of synaptic excitability and plasticity. This raises questions about the potential effects of altered Kir4.1 expression during diverse, natural patterns of neural activity.

In principle, Kir4.1 overexpression, like any other common experimental intervention (e.g., conditional knockouts or sensor expression) could produce some off-target or compensatory effects. However, we estimate that only 5%–10% of astrocytes overexpressed Kir4.1 in our experimental settings. This suggests minimal, if any, impact on developmental homeostasis.

The study also encountered technical limitations, including: (1) a relatively low success rate in experiments tracing individual axons within the territories of labeled astrocytes, (2) insufficient optical resolution to discern the local astrocyte environment of individual axons, (3) an inability to control the cellular sparsity or level of Kir4.1 overexpression, and (4) the current infeasibility of performing single-cell axon-tracing experiments *in vivo*. As with any research study, while the present findings address key questions, they also expand the horizon of scientific inquiry, paving the way for future investigations.

## Resource Availability

### Lead contact

Further information and requests for resources and reagents should be directed to and will be fulfilled by the lead contact, Dmitri Rusakov (d.rusakov@ucl.ac.uk).

### Materials availability

This study did not generate new unique reagents.

## Star★Methods

### Key Resources Table

**Table:** 

REAGENT or RESOURCE	SOURCE	IDENTIFIER
Antibodies		
Rabbit anti-Kir4.1	Proteintech	Cat#12503-1-AP; RRID:AB_2234144
Goat anti-Rabbit IgG (H + L) Cross-Adsorbed Secondary Antibody, Alexa Fluor™ 488	Thermofisher	Cat# A-11008; RRID: AB_143165
Bacterial and virus strains		
AAV9.pZac2.1-gfaABC1Dpromoter> tdTomato:rKcnj10	Vector Builder	Cat# VB171121-1235ysj
AAV9.pZac2.1-gfaABC1Dpromoter> tdTomato	Vector Builder	Cat#VB210711-1154wtd
AAV9.hSynap.iGluSnFR.WPRE.SV40	Penn Vector Core	Cat#Addgene 98929-AAV9
Biological samples		
C57BL/6 Mice	Charles River Laboratories	RRID: MGI:2159769
Chemicals, peptides, and recombinant proteins		
K^+^ sensor GINKO2	Prof. RE Cambell https://doi.org/10.1371/journal.pbio.3001772	pBAD-GINKO2, Addgene Cat#177116
Critical commercial assays		
Oregon Green™ 488 BAPTA-1, Hexapotassium Salt	ThermoFisher	Cat#O6806
Cal-590™, potassium salt	Stratech	Cat#20518
Oregon Green™ 488 BAPTA-1, AM	ThermoFisher	Cat#O6807
Deposited data		
K+ dynamics simulation model	In-house, GitHub depository	https://github.com/RusakovLab/CellReport
Experimental models: Organisms/strains		
C57BL/6 Mice	Charles River Laboratories	RRID: MGI:2159769
Software and algorithms		
MES v4.x-v.6.3	Femontics Ltd.	RRID:SCR_018309
MESc 3.5.7	Femontics Ltd.	https://femtonics.eu/femtosmart-software/
Axon PClamp	Molecular Devices	RRID:SCR_011323
Fluorescent Imaging Analysis Software (FIMAS)	https://github.com/zhengkaiyu/FIMAS	RRID:SCR_018311
Spike2	Cambridge Electronic Design	RRID:SCR_000903
OriginPro	OriginLab Inc.	RRID:SCR_014212
ImageJ	ImageJ	RRID:SCR_003070
Simulation platform BRAINCELL	In-house	www.neuroalgebra.net
Simulation platform ASTRO	In-house	https://modeldb.science/243508.
Other		
Multiclamp 700B	Molecular Devices	RRID:SCR_018455
Digidata 1550	Molecular Devices	Digidata 1550
Femto2D Multiphoton scanning microscope	Femontics Ltd.	Femto2D
Femtosmart microscope	Femontics Ltd.	Femto2D
Leica VT1200S vibrating microtome	Leica BioSystems	RRID:SCR_020243
Mobile HomeCage	Neurotar	Cat#NRT000251-06

## Experimental Model And Study Participant Details

### Animal experimentation

All animal procedures were conducted in accordance with the European Commission Directive (86/609/EEC), the United Kingdom Home Office (Scientific Procedures) Act (1986) with project approval from the Institutional Animal Care and Use Committees of the University College London. All animals were maintained in controlled environments as mandated by national guidelines, on 12hr light/dark cycles, with food and water provided ab libitum.

### Experimental model designs across preparations

For *ex vivo* electrophysiology and imaging both male and female C57BL/6 J mice (Charles River Laboratories) were used. For experiments requiring viral-mediated expression male and female wild-type C57BL/6 mice (Charles River Laboratories) were injected at P0-1 day of age with viral vectors, and acute brain slices were obtained on average four weeks later. For experiments *in vivo*, male and female wildtype C57BL/6 mice (Charles River Laboratories) were injected with viral constructs at 1–1.5 month old; all animals underwent craniotomy and the implantation of a head plate at 4 to 8-week post-injection, as detailed below.

## Method Details

### Viral transduction of genetically encoded sensors

To achieve astrocytic transduction, we employed adeno-associated virus (AAV) vectors expressing either TdTomato-tagged Kir4.1 channel (AAV9.pZac2.1-gfaABC1Dpromoter>TdTomato:rKcnj10, supplied by Vector Builder, CA, USA) or TdTomato alone (AAV9.p-Zac2.1-gfaABC1Dpromoter>TdTomato, supplied by Vector Builder, CA, USA). For imaging of individual boutons, a virus expressing the neuronal optical glutamate sensor, (AAV9.hSynap.iGluSnFR.WPRE.SV40, supplied by Penn Vector Core, PA, USA) was used. All viral vectors were aliquoted and stored at −80°C until use.

For intracerebroventricular (ICV) viral gene delivery, neonatal pups, both male and female (P0-P1), were prepared for aseptic surgery and received ICV injection as described previously.^48^ Viral particles were injected in a volume 2.5 μL/hemisphere (totalling 5 × 10^9^ genomic copies), using a glass Hamilton microsyringe at a rate not exceeding of 0.2 μL/s, 2 mm deep, perpendicular to the skull surface, guided to a location approximately 0.25 mm lateral to the sagittal suture and 0.50–0.75 mm rostral to the neonatal coronary suture. Once delivery was completed, the microsyringe was left in place for 20–30 s before being retracted. Pups (while away from mothers) were continuously maintained in a warm environment to eliminate the risk of hypothermia in neonates. After animals received viral infections, they were returned to the mother in their home cage. Pups were systematically kept as a group of litters. Every animal was closely monitored for signs of hypothermia following the procedure and for days thereafter, to ensure that no detrimental side effects appear. Approximately four weeks were sufficient for the viral transduction to enable satisfactory imaging in acute hippocampal slices. We estimated that, in our settings, only 5–10% of astrocytes overexpressed Kir4.1, thus arguing against any significant compensatory or off-target effects on the overall developmental processes in the brain.

### Hippocampal slice preparation

Transverse hippocampal slices (350 mm thick) were prepared from 3- to 4-week-old mice. The hippocampal tissue was sliced in an ice-cold slicing solution containing (in mM): sucrose 75, NaCl 87, KCl 2.5, CaCl_2_ 0.5, NaH_2_PO4 1.25, MgCl_2_ 7, NaHCO_3_ 25, and D-glucose 25 and left to recover for 20 min in the same solution at 34°C. Then slices were incubated at 34°C in solution containing (in mM): NaCl 119, KCl 2.5, NaH2PO4 1.25, MgSO4 1.3, CaCl2 2.5, NaHCO3 25, and D-glucose 11. Slices were allowed to rest for 1 h before the recordings started. For recordings, slices were transferred to a recording chamber mounted on the stage of an Olympus BX51WI upright microscope (Olympus, Tokyo, Japan) and superfused at 32–34°C. All solutions were saturated with 95% O_2_ and 5% CO_2_. Osmolarity was adjusted to 298 ± 3 mOsM.

### Electrophysiological recordings *ex vivo*

Electrophysiological examination of astrocytes was carried out as previously described.^5,6^ Briefly, whole-cell recordings in astrocytes were obtained using standard patch pipettes (3–4 MΩ) filled with potassium methane sulfonate solution (KMS) based intracellular solution containing (in mM): CH_3_KO_3_S 135, HEPES 10, MgCl_2_ 4, disodium phosphocreatine 10, Na_2_ATP 4, NaGTP 0.4 (pH adjusted to 7.2 with KOH; osmolarity to 290 ± 3 mOsM). Passive astrocytes were identified by their small soma size (~10 μm), low resting potential (below −80 mV), low input resistance (<25 MΩ), passive (ohmic) properties and characteristic morphology of the arbor (Figures 1B and S1). Membrane resistance values were corrected for the patch pipette resistance. Synaptic responses were evoked by single and burst stimulation of Schaffer Collaterals with a bipolar electrode (FHC, Bowdoin, USA). The stimulating electrode was placed in the stratum radiatum, and the astrocytes (normally located 100–300 μm away unless shown otherwise) were either held in voltage clamp mode at their resting membrane potential or in current clamp. In recordings evaluating glutamate transporter current, 50 μM DL-TBOA was added at the end of the experiment, and residual current was subtracted from the total astrocytic current response to isolate glutamate transporter current (IGluT) as described before.^6^

To induce long-term potentiation at CA3-CA1 synapses, an extracellular recording pipette was placed within the territory of the TdTomato labeled astrocytes. Synaptic responses were evoked by Schaffer Collaterals stimulation using a bipolar stimulation electrode placed in the stratum radiatum at >200 μm from the recording site. Field EPSPs (fEPSPs) were recorded using a standard patch pipette filled with the extracellular solution. The baseline stimulus intensity was set at ~50% of the maximal response, control stimuli were applied every 60 s, for at least 10 min to ensure stable responses, before LTP was induced using three trains of high-frequency stimulation (HFS, 100 pulses at 100 Hz) 60 s apart. The slope of fEPSPs was monitored afterward for at least 30 min.

### Immunohistochemistry

Mouse injected with AAV9.pZac2.1-gfaABC1Dpromoter>TdTomato:rKcnj10 AAV was anesthetized with isoflurane and culled via cardiac perfusion with phosphate-buffered saline (PBS). Brains were then post-fixed in 4% paraformaldehyde (PFA; ChemCruz, sc-281692) in PBS for 24 h, transferred to 0.01% sodium azide PBS (Sigma, 71289) after that, and stored at 4°C. Coronal brain sections were cut at 50μm thickness with a Vibrotome (Leica, VT1200). Sections containing hippocampal regions were selected for staining. Sections were stained free-floating for Kir4.1 protein. Tissue sections were permeabilised using 0.3% Triton X-100 PBS for 20 min followed with a blocking agent: natural goat serum (NGS; Invitrogen, 01–6201; 4% NGS in 0.3% Triton X-100 PBS) for 20 min at room temperature. Sections were then incubated with primary antibody: rabbit anti-Kir4.1 IgG polyclonal antibody (1:500; Proteintech, 12503-1-AP) in 0.4% NGS and 0.3% Triton X-100 overnight at 4°C. Sections were washed with PBS and incubated with the secondary antibody goat anti-rabbit IgG (H + L) Alexa Fluor 488 (1:750, Thermofisher, A-11008) in PBS for 2 h at room temperature. The sections were rinsed with PBS, mounted onto slides, and covered with coverslips using Fluoromount-G (Invitrogen, 00-4958-02).

Multiplexed imaging was carried out for the respective red (TdTomato) and green (Alexa) channels using two-photon excitation at λ_x_^2p^ = 940 nm unless specified otherwise.

### Monitoring intracellular [Ca^2+^] using FLIM

Two-photon excitation by femtosecond infrared laser pulses was used to restrict excitation and emission collection to a thin (~0.9 μm thick) focal excitation plane 50–110 μm deep into the slice. The imaging system was based on the Femto2D microscope equipped with a Becker and Hickl FLIM detector (Femtonics, Budapest). The two-photon laser source was a Newport-Spectraphysics Ti:Sapphire MaiTai laser pulsing at 80 MHz, with a pulse width of ~220 fs and a wavelength of either 800 nm or 910 nm for OGB-1 or Cal-590 excitation, respectively. The laser power was kept below 8 mW under the objective, to minimize phototoxic damage. The OGB-1 and Cal-590 lifetime sensitivity to [Ca^2+^] was calibrated as described in previous studies.^55,59^

For astrocytic Ca^2+^ measurements, cell-impermeable OGB-1 (200 μM) was added to the astrocyte patch pipette solution. After approximately 30 min, allowing for dye diffusion, images were acquired from neighboring gap-junction astrocytes at a rate of 500 lines per second. These images were stored as 256 × 512 × 512 x n (*t*,*x*,*y*,*T*) data cubes, representing *xy* images with nanosecond delay time (*t*) distribution at each pixel during the frame duration (*T*).

To assess the dynamics of presynaptic axonal Ca^2+^, in one set of experiments, CA3 pyramidal cells held in whole-cell were dialyzed with the bright morphological tracer Alexa Fluor 488 (300 μm) and the red-shifted Ca^2+^ indicator Cal-590 (300 μM). The axon was traced from the cell body toward area CA1, and fluorescent responses to soma-generated spikes were imaged at individual axonal boutons using a spiral (Tornado) linescan (at 1-2k Hz). In other experiments, cell permeable OGB-1 AM (500 μM) was bolus loaded using a glass pipette, following the method described previously.^80^ In brief, the tip of the pipette was inserted into the stratum radiatum area, and the dye-containing solution was injected at a pressure of 30–40 hPa for 10 min. After 1 h of incubation in a recording chamber, Ca^2+^ transients were recorded in response to Schaffer Collateral stimulation (5 × 50 Hz) using spiral-scan mode (0.5–1 kHz). These recordings were obtained from axonal boutons located at least 200 μm away from the site of dye loading and passing through either TdTomato or TdTomato-Kir4.1* astrocytic territories.

### Axon tracing and imaging of glutamate release *ex vivo*

Presynaptic glutamate release signals were imaged at individual boutons of CA3 pyramidal neurons. In one set of experiments, iGluSnFR-expressing Schaffer collateral axons were initially identified based on their typical smooth morphology, regular presence of boutons, general realignment with the *stratum radiatum*, and, critically, their glutamate-sensitive fluorescent responsiveness to afferent stimuli. Individual boutons falling within the TdTom-labelled astrocytic territories were selected. A spiral-line scan was positioned over the bouton of interest, which was then scanned at a sampling frequency of approximately 500 Hz using two-photon excitation at 910 nm. Glutamate release was induced by stimulating the Schaffer collaterals with a paired-pulse protocol (2 stimuli x 20 Hz) with a “minimal” stimulation strength so that glutamate release successes and failures at individual boutons could be readily detected. To ensure robust measurements, at least 15 trials per bouton were recorded. The average release probability (P_r_) was directly calculated by dividing the total number of successful releases by the total number of trials for each experimental condition. The paired-pulse ratio (PPR) was calculated as the corresponding average probability ratio.

In another set of experiments, to directly compare glutamate release parameters within and outside the Kir4.1 astrocytic territory, an *s. radiatum* region that includes such a territory and a control area outside is selected for fast resonant scanning with a FemtoSmart imaging system (Femtonics, Budapest) integrated with patch-clamp. The resonant *XY* scan time series was typically performed as 512 × 50 × 317 pixel frame scan at a rate of 300–330 Hz for 1 s duration. Presynaptic stimulation was induced by stimulating Schaffer collaterals (5 stimuli x 20 Hz), and glutamate release was recorded using resonant frame scanning mode in control conditions under 2.5 mM [K^+^]_out_, during 15–35 trials ~1 min apart. Next, [K^+^]_out_ was elevated to 5 mM, and after 5–7 min washing in, another series of frame scan recordings consisting of 15–35 trials were subsequently collected. The time lapse protocol applied to both imaging channels, and the region of scanning (typically ~120 μm × 12 μm) was checked, refocused on if required, and aligned between the scans to maintain the same focal plane throughout the experiment. To minimise photobleaching, only a single focal section was acquired for imaging at a low laser power (4–6 mW under the objective).

To analyze glutamate release at individual axonal boutons, images collected with MESc software v 3.5.7 (Femtonics, Budapest) were exported as *t*-stacks, averaged between trials, and the 1.5–2.5 μm wide regions of interest (ROIs) were manually selected using ImageJ software for further analyses. The individual ROIs never overlapped and were at least 1.5–2 mm away from its nearest neighbors. The *ΔF/F*_*0*_ value, represented the change in fluorescence relative to the baseline, was then calculated across all ROIs. The PPR value was calculated as the ratio between the 10-ms average peak values of the second and the first fluorescence responses to paired afferent stimuli.

### Viral transduction of astroglial Kir4.1 in the cortex

To express the Kir4.1 channels in cortical astrocytes, unilateral injections of an AAV virus expressing Kir4.1 channels under the GfaABC1D promoter were performed into the somatosensory cortex of C57BL6/N mice in aseptic surgical procedures. Perioperative multimodal analgesia was carried out with buprenorphine (60 μg kg^−1^, s.c.) and lidocaine (2.5%) topically applied to the surgical site; ocular ointment (Lacri-lube, Allergan, UK) was also applied. Isoflurane was used for anesthesia throughout the surgical procedure: 4.5–5% v/v for induction and 1.5–2.5% v/v for maintaining the anesthesia level. Body temperature was maintained at ~37.0°C using a feedback rectal thermometer and heating blanket. After localising bregma in the mouse head fixed in a stereotaxic frame, a local opening through the scull (~1 mm diameter) was made with a high-speed dental drill at the coordinates for the somatosensory cortex (relative to bregma: AP: −1.5 mm, ML: −3 mm). Pressure injections were conducted using a Hamilton syringe stereotactically guided to a depth of 1 mm beneath the cortical surface, under control of a microinjection pump at a rate of approximately 20 nL min^−1^. The total injection volume (300–500 nL) was delivered in three steps, reducing depth by 150–200 μm at each step. Once delivery was completed, a needle was left in place for approximately 5 min before being retracted. The surgical wound was closed, metacam (1 mg kg^−1^, s.c.) and saline (0.5 mL) were administered, and the animal was left to recover in a heated chamber.

### Headplate installation, craniotomy, durotomy

Mice were prepared for craniotomy as described for the viral injection procedure 3–4 weeks after *in vivo* transduction of viral vectors. Once secured and deeply anesthetized, the skull’s right frontal and parietal bones were exposed; the area was cleaned and coated with tissue adhesive (3M Vetbond, UK) to facilitate headplate installation. A custom-made headplate was affixed over the right somatosensory cortex (the targeted injection site) and secured with dental cement (SuperBond, Sun Medical Co. Ltd., Japan). Once the headplate was fixed and the cement components cured, the animal was secured in a custom-built head fixation frame. A craniotomy of ~3 mm diameter was performed over the S1BF region using a high-speed hand drill. After sufficiently thinning the skull and super-fusing its surface with saline, the skull flap was removed using fine-tipped forceps. Immediately after opening, the brain was super-fused with sterile saline. Durotomy was carried out using 28G needles with hand-made curved tips, avoiding penetrating or damaging the pia mater. After completing the surgery, the anaesthetia regime was switched from inhalation to i.p. injection, using a mixture of fentanyl (0.03 mg kg-1), midazolam (3 mg kg-1), and medetomidine (0.3 mg kg-1), for the subsequent imaging in the anesthetized animal.

### Expression and purification of GINKO2 protein

To prepare the GINKO2 protein, a single colony of *E. coli* DH10B transformed with pBAD/His B harboring GINKO2 was used to inoculate 500 mL of Terrific Broth (TB) supplemented with 100 μg/mL ampicillin and 0.02% (w/v) L-(+)-arabinose. The culture was incubated at 37°C with shaking at 220 rpm for 16 h. Protein purification was performed as previously described.^64^ The purified GINKO2 protein was buffer exchanged into 10 mM HEPES (pH 7.4) with PD-10 desalting columns (GE Healthcare Life Sciences) and concentrated with Amicon Ultra-15 Centrifugal Filter Devices. Aliquots of the concentrated protein were flash frozen in liquid nitrogen and stored at −80°C. The protein solution was thawed immediately before use.

### Multiplexed 2PE imaging *in vivo*

An optical probe GINKO2 (6.55 mM) was applied onto the surface of the exposed cortex at a volume of 100 μL for 60–90 min, taking care to fully cover the open brain area using repeated applications if needed. After loading the optical probe, the animal was transferred to the FemtoSmart imaging system (Femtonics, Budapest) integrated with electrophysiology and linked to the two-beam tunable femtosecond pulse laser MaiTai (SpectraPhysics-Newport) for multiplex imaging. The anesthetized animal was secured on a custom-built stage via the installed headplate under XLPlan *N* 25× water immersion objective (NA 1.05) coupled to a green lamp illumination. 2PE acquisitions were performed with laser at 940 nm optimised for GINKO2 for selecting areas with bright GINKO2 signal (green channel) containing TdTomato-positive cortical astrocytes (red channel). Imaging was performed mainly in the L1 layer, at 40–100 μm depths. Imaging settings were adjusted to provide optimal recording conditions, adjusting laser intensity to minimize photobleaching (<15 mW). After identification of a suitable area, frame scans of the selected area (approximately 350 × 250 μm) were performed using galvo scanners at 20-35 Hz to sample the baseline GINKO2 signal for typically 3–5 min. To prevent photodamage over prolonged recordings and to correct for possible focal drifts, the time-lapse changes in GINKO2 fluorescence were acquired in 20-30-s time sections for baseline sampling, followed by immediate acquisition after applying high potassium to evoke CSD. The latter constituted a brief puff of high potassium (1 M) applied near the brain surface using a pressurised micropipette, and the focal plane images were sampled for the next 10–20 min.

For the analysis of the GINKO2 fluorescence kinetics, a series of *t*-stacks were concatenated and aligned for the corresponding time throughout the recording, including baseline and high-potassium-induced CSD periods. Fluorescence values were analyzed for several regions of interest (ROIs) from the same focal plane, both outside and within the TdTomato-positive Kir4.1* astrocytic clouds, avoiding somata. Changes in the GINKO2 signal were expressed as the ratio of the dye fluorescence at the maximum of the fluorescent signal over the baseline (Δ*F*/*F*_0_). The values for each ROI, before and after high-potassium-induced CSD, were averaged to yield relative changes between ROIs within and outside the Kir4.1-overexpressing astrocytes.

### LFP recordings combined with widefield imaging of CSD in awake mice

Surgical procedures were performed following induction of deep anesthesia by 4.5% isoflurane and maintenance of anesthesia using 1.5–2.5% isoflurane. A mixture of buprenorphine hydrochloride (0.5 mg/kg), metacam (15 mg/kg) and saline was injected subcutaneously to provide analgesia. A small burr hole in the contralateral anterior region of the skull was created to insert a support screw. A headplate (Neurotar Model 9) was attached via dental cement (Kemdent Simplex Rapid) and tissue adhesive (3M Vetbond) to the surface of the skull. The inner region of the headplate was filled with a silicon polymer (W.P.I. Kwik-Cast); see^81^ for further information regarding surgical procedures.

Following at least a four-day recovery period, the mice underwent their first habituation session. Animals were head-fixed on the Multicage Training Arena (Neurotar) and allowed to habituate for 15 min. Following a minimum of 15 h, a second habituation was performed for 30 min. Lastly, a third was performed for 60 min.

On the day of experimentation, anesthesia and analgesia was induced and maintained as performed previously. Dexamethasone (Duphacort; 0.5 mL/kg) was injected subcutaneously to minimise craniotomy-induced inflammation. Mice were headfixed in a stereotaxic frame and the previously-placed silicon covering removed. A small hole in the skull above the contralateral cortex (AP = +1.5mm, ML = −1.2mm) was drilled to allow later insertion of an AgCl reference electrode. Following this, a diamond-burr drill was used to outline a craniotomy over the visual and somatosensory cortices. Once the skull was sufficiently thinned around the craniotomy site, forceps were used to remove the loose skull. A silicon polymer was used to protect the region during recovery. The mouse was allowed 2-4 h to recover before experimentation.

Following recovery, mice were head-fixed in the Mobile HomeCage (Neurotar) and the silicon covering removed. Following several small perforations of the dura mater (using a bevelled 28G needle), GINKO2 (6.55 mg/mL) was repeatedly applied to the cortical surface for 60 min. Following incubation, a 1× objective was lowered and focused on the cortical surface. GINKO2 was excited by a 470 nm widefield LED focused by the imaging objective (1.072 mW/cm^2^ Cairn OptoLED). An Ag/AgCl wire was shallowly inserted into the previously-generated small contralateral craniotomy. A glass micropipette was inserted approximately 500 μm into the somatosensory cortex to record LFP (Axon Instruments Headstage; Multiclamp 700B). Images were acquired (Photometrics Evolve 512) with a 20 ms exposure time using Micromanager. A micro1401 connected to Spike2 (Cambridge Electronic Design) was used to record LFP from the glass micropipette. All acquisition was synchronised by the delivery of TTL pulses and recording camera frame exposure. Glass micropipette recordings were continuous through the experiment. Fluorescence dynamics were acquired in 10-min imaging sweeps, with baseline recordings revealing a healthy tissue state. Following baseline recording, injection of KCl (150 nL 1M 50 nL/s; WPI Microsyringe Pump; Hamilton Model 95 Syringe, 33G needle) was performed into the primary somatosensory cortex. The subsequent CSDs were recorded with synchronised electrophysiology and imaging. Recording of activity was performed until 1–2 h post head-fixation. Following this, sodium pentobarbital was injected (i.p.) to terminate the experiment.

### Simulations: Realistic model of a Kir4.1-expressing astrocyte

The simulations used the computational modeling platform ASTRO^26^ and BRAINCELL (www.neuroalgebra.net); the CA1 astrocyte model was based on a 3D-reconstructed main-branch tree populated with stochastically generated nanostructures to reproduce the experimental statistics obtained using 3D electron microscopy samples.^26^ The cell surface-to-volume ratio varied form 7 μm^−1^ near the cell body to ~22 μm^−1^ on average toward the periphery, to match experimental observations. Detailed settings for K+ diffusion and related mechanisms are described and enabled in ASTRO/BRAINCELL menus (www.neuroalgebra.net).

The typical CA1 astrocyte surface area of ~20,000 μm^226^and the measured Kir4.1 current *I*_*Kir*_ ~1 nA (at V_m_ = −80 mV) give the Kir4.1 current density ~5 mA/cm^2^, or conductance density *g*_*Kir*_ = 0.175 mS/cm^2^. The latter was increased to *g*_*Kir*_ = 0.35 mS/cm^2^ for the Kir4.1-overepxressing astrocyte or reduced to 0.087 mS/cm^2^ for the Kir4.1-’under-expressing’ cell. The Kir4.1 current kinetics followed the formula obtained by analysing and replicating experimental recordings^26,34^

$I_{Kir\;}=g_{Kir\;}\cdot V_{f}\left(V_{1}\right)\cdot {\left[K^{+}\right]}_{out\;}^{1/2}{\left(1+{\text{e}}^{V_{f}\left(V_{2}\right)/V_{3}}\right)}^{-1/2}$ where *V*_*f*_ (*V*_*i*_) = *V*_*m*_ − *A*_*K*_ *E*_*K*_ − *V*_*i*_, *V*_*m*_ is membrane potential, *V*_*1*_= −14.83 mV, *V*_*2*_ = −105.82 mV, and *V*_*3*_ = 19.23 mV are best-fit parameters for experimental *I*_*Kir*_ recordings^34^ and normalization factor *A*_*K*_ = 0.6891 accounts for *I*_*Kir*_ = 0 at [K^+^]_out_ = 2.5 mM and *V*_*m*_ = −80 mV. The reversal potential for potassium *E*_*K*_ was calculated using the Nernst equation $E_{K}=\frac{RT}{F}\log\frac{{\left[K^{+}\right]}_{out\;}}{{\left[K^{+}\right]}_{in\;}}$ where [K^+^]_in = _ 110 mM, [K^+^]_out_ is variably adjusted, gas constant *R* = 8.31 J M^−1^·C^−1^, *T* is absolute temperature, and the Faraday’s constant *F* = 96485 C M^−1^.

### Simulations: 3D dynamic landscapes of [K^+^]_out_ and [K^+^]_in_

The realistic 3D astrocyte model equipped with the Kir4.1 channels and other membrane mechanisms as described above, was placed at the center of the simulation arena. The computation of ion extracellular dynamics was carried out using BRAINCELL (www.neuroalgebra.net) which incorporated the NEURON RxD arrangement (). The simulations utilized the CA1 astrocyte geometry from the BRAINCELL catalogue, implemented using the file ‘AstrocyteBasicGeometry.hoc’. The overall arena dimensions were 130 × 150 × 42 μm^3^, centered at (2, −1, −3) μm. The ion dynamics in the extracellular space was computed using a 3D grid with an x-y-z resolution of 19 × 28 × 15 steps. The boundary condition was Neumann’s (zero flux). The selected network dimensions provided an optimal balance between computational efficiency and minimal edge effects, with boundary conditions influencing [K^+^]_out_ dynamics by less than 5% in the vicinity of the astrocyte. The initial [K^+^]_out_ throughout was [K^+^]_out,0_ = 5 mM, which decayed to the equilibrated (steady state) level of 2.5 mM, according to the formula: $\frac{d{\left[K^{+}\right]}_{out\;}(r,t)}{\partial t}=\tau^{-1}\left({\left[K^{+}\right]}_{out\;}(r,t)-{\left[K^{+}\right]}_{out\;,ss}\right)$ where **r** is the voxel co-ordinates, [K^+^]_out,ss_ = 2.5 mM, and τ is the time constant. This formula at τ = 40 s represents a first-order approximation of the simulated [K^+^]_out_ around a typical astrocyte, which hosts Kir4.1 at a conductance density of *g*_*Kir*_ = 0.175 mS/cm^2^ and ~20,000 μm^2^ total cell membrane area (corresponding to Kir4.1 current of ~1 nA at V_m_ = -80 mV), occupying ~10% tissue volume fraction.^5,26^ At the voxels representing the central astrocyte, the Kir4.1 conductance density was set either at the additional *g*_*Kir*_ = 0.175 mS/cm^2^ (giving the total of *g*_*Kir*_ = 0.35 mS/cm^2^) or at negative *g*_*Kir*_ = −0.087 mS/cm^2^ (giving the total of *g*_*Kir*_ = − 0.087 mS/cm^2^), to represent a Kir4.1* or Kir4.1-under-expressing central astrocyte, respectively. The astrocyte model incorporated a passive conductance *g*_pas_ = 0.001 mS cm^−2^ and reversal potential *E*_*K*_ = −80 mV to maintain stable membrane depolarization.

Correspondingly, the [K^+^]_in_ dynamics was computed, across individual voxels of the central astrocyte, according to the formula *[K*^+^]_*in*_(**r**; *t*) = 2$([*K*^+^*]*_*out*;0_ – [*K*^+^*]*_*out*_(**r**; *t*)) which ‘mirrors’ the local [K^+^]_out_ dynamics, with factor 2 reflecting the average ratio ‘extracellular/intracellular volume fraction’ for CA1 astrocytes^5,26^. The extracellular diffusion coefficient for K^+^, including space tortuosity, was 0.6 μm^2^ ms^−1^; small-scale intracellular diffusion equilibration in these simulations was ignored. The simulations were performed with a 1 ms numerical integration time step over a total duration of 20 s. In all simulations we assumed uniform Kir4.1 distributions across cell membranes or tissue volume.

## Quantification And Statistical Analysis

The experiments involved were generally technically complex and/or time consuming. Therefore, throughout our *ex vivo* experiments, each sampled value (graph points/circles illustrating sample size *n*) corresponded to one cell from one brain slice, with 1-2 slices used per animal, unless specified otherwise. Electrophysiological data were analyzed with WinWCP and Clampfit (Axon Instruments Inc.; Union City, USA). Imaging data were analyzed using MES and MESc v 3.5.7 software (Femtonics, Budapest), ImageJ (a public domain Java image processing program by Wayne Rasband), and traces expressed as D*F/F*_*0*_. Statistical analyses were performed using Excel (Microsoft, US) or Origin 2023 (OriginLab Corp, Northampton, USA). Shapiro-Wilk tests for normality were routinely run for small samples; this test for the means could be misleading for *n* > 15–19 due to Central Limit Theorem. Correspondingly, two-tailed paired and unpaired Student’s *t*-test, or otherwise non-parametric Mann-Whitney tests were used for statistical analyses. Mean difference was considered significant at the null-hypothesis rejection level of *p* < 0.05. Statistical summary data are shown as mean ± SEM unless specified otherwise. To account for the factors of slices versus recorded cell pairs, a Kruskal-Wallis non-parametric ANOVA was performed. No significant difference in the recorded data metrics was detected between preparations from male and female animals in our settings, so the data were pooled.

## Supplementary Material

Supplemental information can be found online at https://doi.org/10.1016/j.celrep.2025.115299.

Supplementary figures

## Data Availability

Computational platform BRAINCELL for detailed biophysical modeling of astrocytes (and other brain cells) and their environment is freely available on www.neuroalgebra.net. The programming codes pertinent to the simulations of K+ dynamics inside and outside astrocyte are deposited at and available freely at the NEURON database: 243508, https://modeldb.science/243508, and additionally at https://github.com/RusakovLab/CellReport. The original codes are publicly available as of the date of publication. Further data reported in this paper will be shared by the lead contact upon request. Any additional information required to reanalyze the data reported in this paper is available from the lead upon request.

## In brief

Tiurikova et al. show that the astrocyte channels Kir4.1 set the level of activity-dependent local excitation by regulating presynaptic Ca^2+^ dynamics and glutamate release at excitatory synapses.

## Highlights

- Kir4.1 expression sets the speed of glutamate and potassium uptake by astrocytes
- Short-term presynaptic plasticity varies with Kir4.1 expression at local astrocytes
- Increased astrocyte enrichment in Kir4.1 attenuates cortical spreading depolarization
- Individual astrocytes can internally dissipate K^+^ during moderate neural activity
