# Supplementary material for: Astrocyte Kir4.1 expression level territorially controls excitatory transmission in the brain
Source: Cell Rep. Author manuscript; Available in PMC 2025 Nov 24. (PMC7618397; doi:10.1016/j.celrep.2025.115299)
Supplement: Supplementary figures [file EMS210860-supplement-Supplementary_figures.pdf]

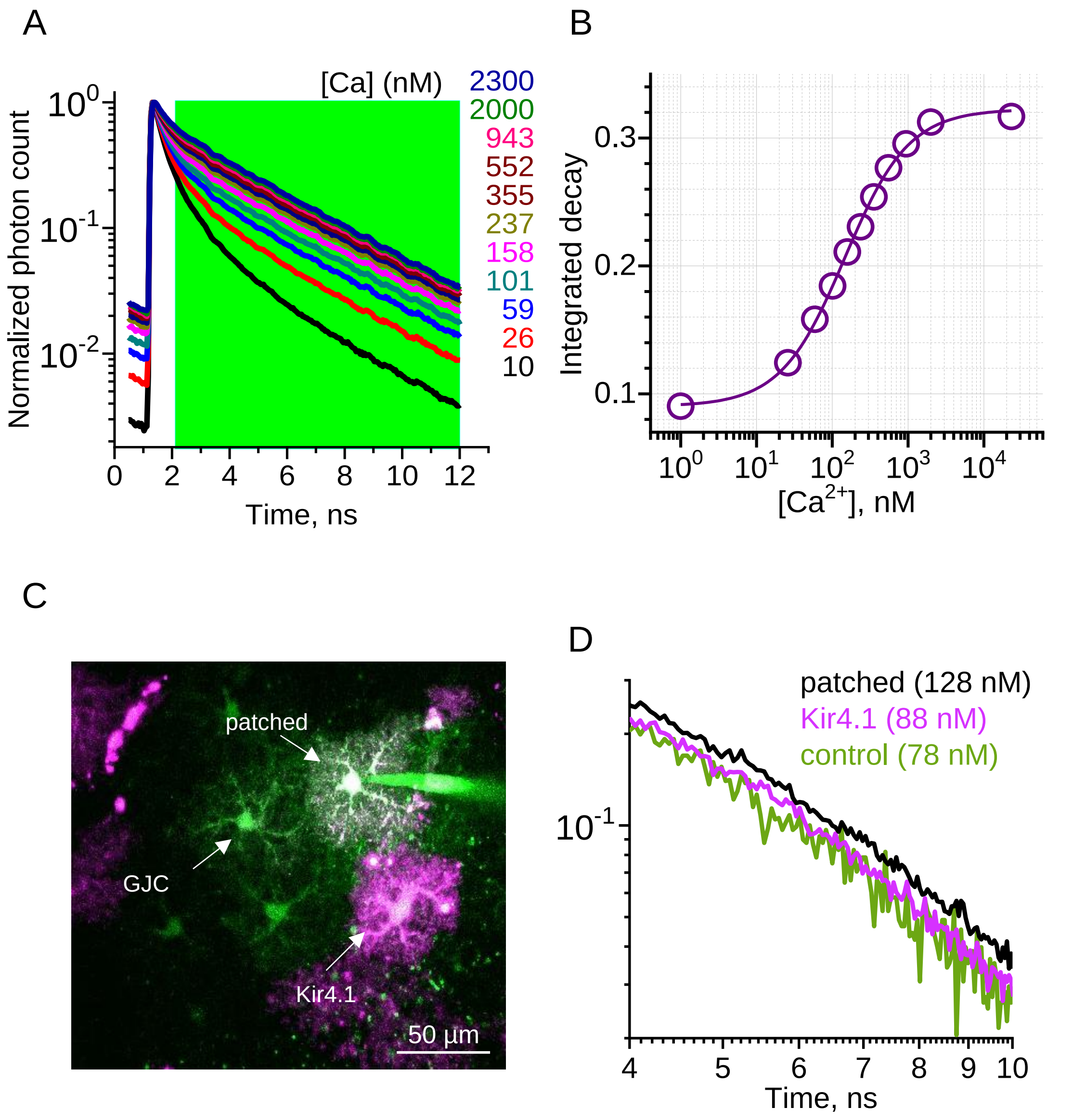

Figure S1. FLIM calibration of OGB-1 for its lifetime sensitivity to [Ca^{2+}].

(A) Fluorescence lifetime of OGB-1 in calibrated solutions of clamped [Ca^{2+}]; concentrations are shown in nM; green shade shows the time range for 'area-under-the-curve' calculation.

(B) Summary calibration curve plotted as normalised total photon count calculated as the ratiometric measure 'area-under-the-curve / peak' value of the fluorescence lifetime (integrated decay) plotted against [Ca^{2+}]; circles, individual data points; line, logistic best fit; see Ref [56] for detail.

(C) Image: illustration as in Figure 2A, with explanatory notes.

(D) Graph, fluorescence lifetime of OGB-1 for patched, Kir4.1* and tdTom (control) astrocytes: a fragment from Figure 2C plot expanded for clarity.

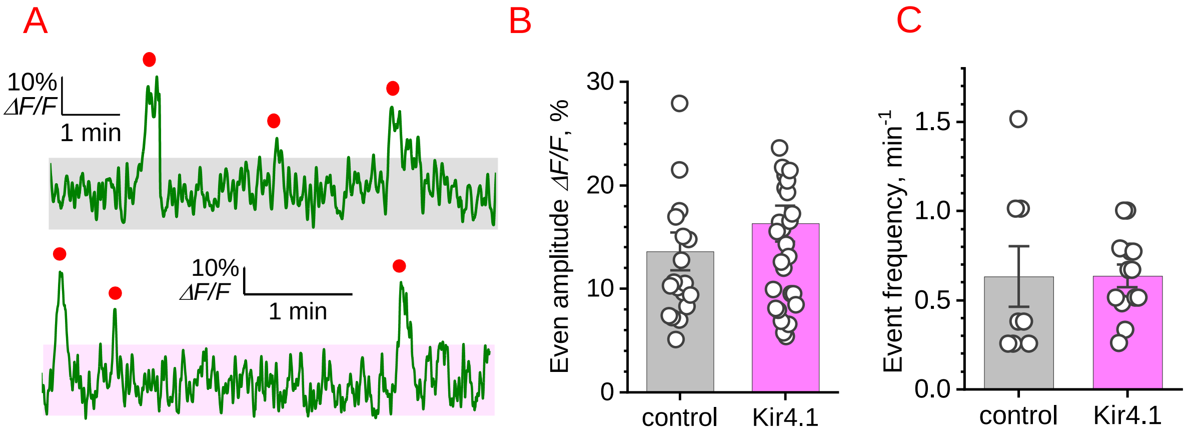

Figure S2. Spontaneous Ca^{2+} signals in control and Kir4.1 astrocytes have similar occurrence rates and amplitudes.

(A) Examples of representative trace fragments depicting spontaneous Ca^{2+}-sensitive OGB-1 fluorescence signals within the soma of control (top) and Kir4.1* (bottom) astrocytes, as indicated; shaded areas, the amplitude range (three standard deviations of the background noise) above which the events are considered significant; red dots, registered Ca^{2+} events.

(B) The Ca^{2+} event amplitudes (mean ± SEM) in control and Kir4.1* astrocytes, as indicated; n = 19 and n = 29, respectively; circles, individual events recorded from 8 and 13 cells, respectively.

(C) The frequency of Ca^{2+} event (mean ± SEM) in control and Kir4.1* astrocytes, as indicated; circles, individual cells; n = 8 and 13, respectively.

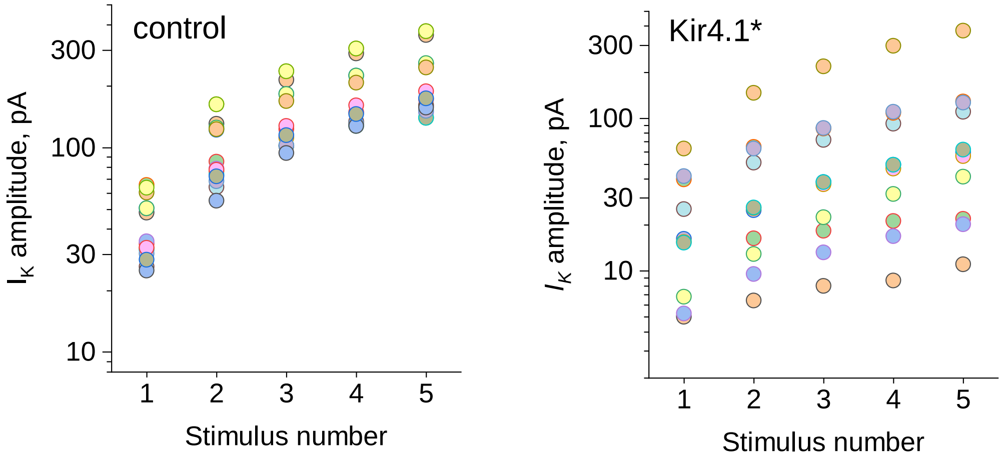

Figure S3. Amplitudes of stimulus-evoked hole-cell K^+ currents recorded from control and Kir4.1* astrocytes.

Summary of the absolute amplitudes of potassium current (I_K) recorded in control (left, n = 15) and Kir4.1* (right, n = 11) astrocytes.

Note that, because of a wider and more favourable experimental sampling of control (WT) astrocytes with respect to the stimulating electrode, the stimulus-evoked currents were consistently larger in control compared with Kir4.1* astrocytes.

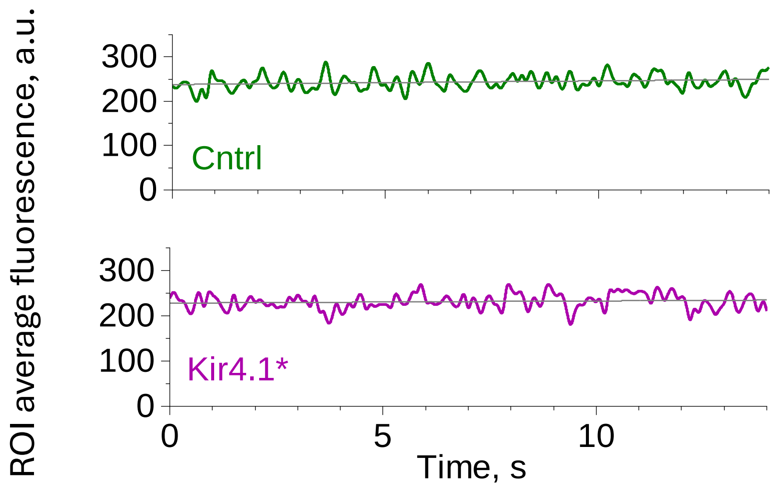

Figure S4. Fluorescence stability of extracellular GINKO2 following its bath washout.

An example of the average fluorescence level of extracellular GINKO2 within the territory of control (top) and Kir4.1* (bottom) astrocyte, recorded continuously over a 14 min period. Straight lines depict linear regression of the experimental data.

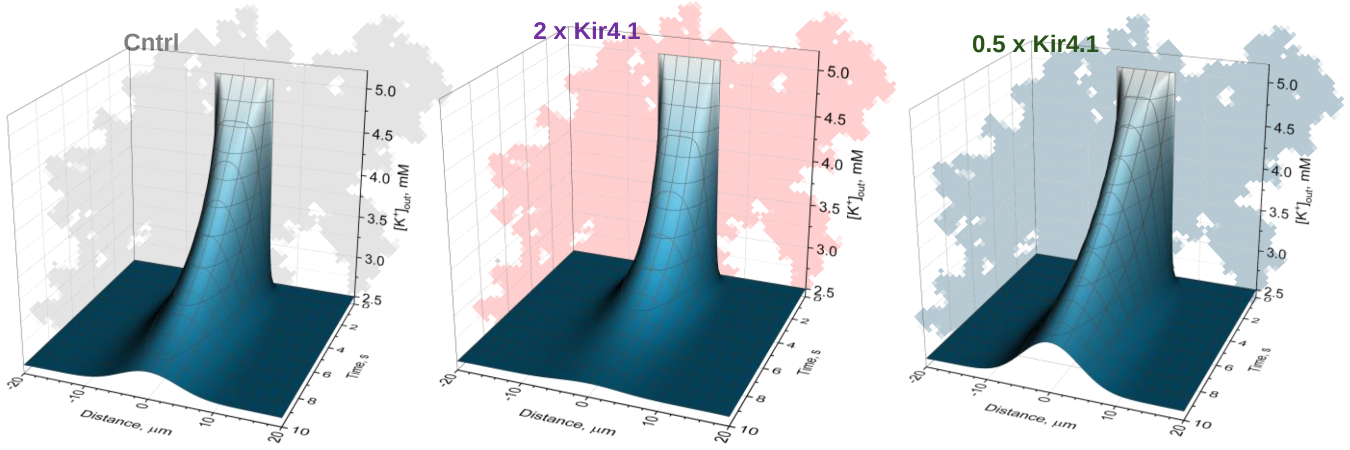

Figure S5. Kir4.1 expression regulates local sink of [K$^+$]out: biophysical simulations.

The dynamic landscape of [K$^+$]$_{out}$ over 10 s following a quasi-instantaneous increase of [K$^+$]$_{out}$ in a local area (10 µm sphere centred at the astrocyte centroid), from resting 2.5 mM to 5 mM, as in Figure 6A, as sampled in a cross-section through the centre of either a control (left, grey astrocyte shape), Kir4.1-overepxressing (centre, 2 x Kir4.1, red shape), or Kir4.1-underepxressing (right, 0.5 x Kir4.1; teal shape) astrocyte.
